# Reactivation of NR4A1 Restrains Chondrocyte Inflammation and Ameliorates Osteoarthritis in Rats

**DOI:** 10.3389/fcell.2020.00158

**Published:** 2020-03-17

**Authors:** Yan Xiong, Jisheng Ran, Langhai Xu, Zhou Tong, Moqbel Safwat Adel Abdo, Chiyuan Ma, Kai Xu, Yuzhe He, Zhipeng Wu, Zhonggai Chen, Pengfei Hu, Lifeng Jiang, Jiapeng Bao, Weiping Chen, Lidong Wu

**Affiliations:** ^1^Department of Orthopedic Surgery, The Second Affiliated Hospital, School of Medicine, Zhejiang University, Hangzhou, China; ^2^Department of Medical Oncology, The First Affiliated Hospital, Zhejiang University School of Medicine, Hangzhou, China

**Keywords:** osteoarthritis, NR4A1, NF-κB signal pathway, histone deacetylase, mitogen-activated protein kinase, cytosporone B

## Abstract

Osteoarthritis (OA) is the most prevalent joint disease and uncontrolled inflammation is now recognized to play vital roles in OA development. Targeting the endogenous counterpart of inflammation may develop new therapeutic approaches in resolving inflammation persistence and treating inflammatory disease including OA. The orphan nuclear receptor 4A1 (NR4A1) is a key negative regulator of inflammatory responses but its role in osteoarthritis remains unclear. In the present study, we found that the NR4A1 expression was elevated in human osteoarthritis cartilage and *in vitro* OA model, which could be blocked by NF-κB signal inhibitor JSH23. The overexpression of NR4A1 inhibited, whereas knockdown of NR4A1 enhanced IL-1β induced COX-2, iNOS, MMP3, MMP9 and MMP13 expression, and luciferase reporter activity of NF-κB response element. Though NR4A1 was upregulated in inflammatory stimulation and creates a negative feedback loop, persistent inflammatory stimulation inhibited NR4A1 expression and activation. The expression of NR4A1 declined rapidly after an initial peak in conditions of chronic IL-1β stimulation, which could be partially restored by HDACs inhibitor SAHA. The phosphorylation of NR4A1 was increased in human osteoarthritis cartilage, and p38 inhibitor SB203580, JNK inhibitor SP600125 and ERK inhibitor FR180204 could significantly inhibited IL-1β induced NR4A1 phosphorylation. Reactivation of NR4A1 by its agonist cytosporone B could inhibit IL-1β induced chondrocyte inflammation and expression of COX-2, iNOS, MMP3, MMP9, and MMP13. In rat OA model, intra-articular injection of cytosporone B protected cartilage damage and ameliorated osteoarthritis. Thus, our study demonstrated that the NR4A1 is a key endogenous inhibitor of chondrocyte inflammation, which was relatively inactivated under chronic inflammatory stimulation through HDACs mediated transcriptional suppression and MAKP dependent phosphorylation in osteoarthritis. NR4A1 agonist cytosporone B could reactivate and restore the inhibitory regulatory ability of NR4A1, prevent excessive inflammation, and ameliorates osteoarthritis.

## Introduction

Osteoarthritis (OA) is the most prevalent joint disease and a leading cause of disability worldwide, affecting an estimated 10% of men and 18% of women over 60 years of age (Woolf and Pfleger, 2003). OA is characterized as progressive articular cartilage loss and remodeling of the underlying bone in the synovial joints (Goldring and Goldring, 2016). Currently, OA treatments mainly consist pain management, viscosupplementation and joint replacement, which all focus on the symptoms of advanced disease and no effective approaches available to inhibit the processes that drive OA pathology (Roos and Arden, 2015).

Osteoarthritis was once viewed as a typical non-inflammatory disease of purely mechanical cartilage degradation. Recent studies have evidenced that OA is a complex condition affecting the entire joint structure, in which prolonged inflammation and matrix proteases activation play a pivotal role (Liu-Bryan and Terkeltaub, 2015; Robinson et al., 2016; Scanzello, 2016; Berenbaum et al., 2017). A physiological inflammatory response, which is limited and localized, is beneficial in tissue repair and homeostasis restoration (Medzhitov, 2008). However, the uncontrolled chronic inflammation always results in tissue destruction and the development of inflammatory disease (Medzhitov, 2008; Afonina et al., 2017; Rathinam and Chan, 2018). Though the precise mechanism is not fully understood, the dysfunction of negative autoregulatory loops and the inactivation of the endogenous counterpart are thought to contribute significantly to onset of the runaway inflammation (Gilroy and De Maeyer, 2015; Sugimoto et al., 2016). Targeting the endogenous counterpart of inflammation may develop new therapeutic approaches in resolving inflammation persistence and treating inflammatory disease including OA (Alessandri et al., 2013).

The orphan nuclear receptor 4A1 (NR4A1, also known as NUR77, TR3, NGFI-B, or NAK-1), a member of the NR4A subfamily of nuclear receptors, is increasingly recognized as a key regulator of inflammatory responses and its downstream nuclear factor κB(NF-κB) pathway (Rodriguez-Calvo et al., 2017). The expression of NR4A1 can be rapidly induced by a variety of inflammatory stimuli through nuclear NF-κB pathway activation in monocytes and macrophages (Pei et al., 2005). The activated NR4A1 can in turn inhibit NF-κB activation via impairing p65 binding to DNA (Li et al., 2015) and directly inducing other NF-κB inhibitors expression like IκB (You et al., 2009). Although NR4A1 acts as a natural counterpart of inflammatory response in physiological conditions, it’s always down-regulated and inactivated in inflammatory and chronic diseases like neuroinflammation and fibrosis (Palumbo-Zerr et al., 2015; Shaked et al., 2015). Correcting the dysfunction of NR4A1 provided promising results in restraining uncontrolled inflammation and improving inflammatory disease outcomes.

In the present study, we demonstrated the NR4A1 as a key negative regulator for controlling NF-κB pathway in chondrocytes and osteoarthritis. Though the inflammatory stimulation induced NR4A1 expression, long-time stimulation, however, inactivated NR4A1 through HDACs dependent epigenetic slicing and mitogen-activated protein kinase (MAPK) dependent phosphorylation, resulting in the prolonged inflammatory response in chondrocytes. Reactivation of NR4A1 by its specific agonist Cytosporone B can rebalance chondrocyte inflammatory response *in vitro* and ameliorate osteoarthritis *in vivo*. Our study partially explained the mechanism of chronic chondrocyte inflammation in osteoarthritis and demonstrated NR4A1 as a potential therapeutic target for OA treatment.

## Materials and Methods

### Human Sample

This study was in accordance with the Helsinki Declaration of 1975, as revised in 2000 and approved by the Ethics Committee of The Second Affiliated Hospital, School of Medicine, Zhejiang University, Hangzhou, China. Femoral head cartilage from ten osteoarthritis patients and ten femoral neck fracture patients (as normal control) who received total hip arthroplasty (THA) were collected.

### Chondrocyte Isolation, Culture and Treatment

Knee cartilage harvested from 4-week-old Sprague Dawley rats was cut into 1-mm3 particles and immediately digested with 0.25% pancreatic enzymes for 30 min followed by 0.2% collagenase II on a horizontal shaker at 37°C for 4 h. The isolated chondrocytes were then filtered, centrifuged, resuspended and seeded in 25cm^2^ flasks. The chondrocytes were grown in low-glucose DMEM (Dulbecco’s modified Eagle’s medium):F12 1:1 with 10% FBS (fetal bovine serum), 100U/mL penicillin, and 100mg/mL streptomycin (all purchased from Gibco) at 37°C with 5% CO_2_. Chondrocytes were passaged when grew to approximately 90% confluence, and less than passage 3 were used in our study.

Before stimulation, chondrocytes were treated with serum free medium containing 2 mM L-glutamine (both purchased from Lonza) for 16 h. Where indicated, cells were then incubated for an additional hour in the presence of different reagents before stimulation with 10 ng/ml recombinational interleukin 1 beta (IL-1β) (R&D Systems). For protein phosphorylation level detections, IL-1β treating time was half an hour before harvesting. For lone time stimulation experiments, IL-1β treating time points were 15 min, 30 min, 1, 2, 4, 8, 24, 48, and 72 h. For nuclear protein detection, IL-1β treating time was 3 h. Otherwise, IL-1β treating time was 16–24 h. The treated cells were then lysed with either RIPA Lysis Buffer (BeyondTime) or RNAiso Plus (TAKARA) according to product instructions for western-blot analysis or real time polymerase chain reaction (RT-PCT), respectively. NF-κB inhibitor JSH23, pan-HDACs inhibitor suberoylanilide hydroxamic acid (SAHA), p38 inhibitor SB203580, JNK inhibitor SP600125 and ERK inhibitor FR180204 were obtained from selleck and the concentrations were all 10 μM except for SAHA (5 μM) in our study. NR4A1 agonist Cytosporone B was obtained from Santa cruz.

### RNA Extraction and RT-PCR

Total RNA was isolated by a one-step phenol chloroformeisoamyl alcohol extraction as described by the manufacturer’s protocol. Real-time PCR analysis of genes was performed using Brilliant SYBR Green QPCR Master Mix (TakaRa) with a Light Cycler apparatus (ABI STEPONE PLUS), as described previously (Yin et al., 2010). The primer sequences used in this study are listed in Supplementary Table S1. Each real-time PCR run was performed with at least three experimental replicates, and the results are presented as target gene expression normalized to 18S.

### Protein Extraction and Western Blot

Cultured cells or grinded cartilage tissue were lysed for whole protein extraction by RIPA Lysis Buffer containing protease and phosphatase inhibitors (Boster, China). For cytoplasmic and nuclear protein extraction, the Subcellular Nucleus and Cytoplasmic Protein Extraction Kit (Boster, China) was used according to the manufacture’s protocol. The samples were then separated by 10% or 12% sodium dodecyl sulfate (SDS)-polyacrylamide gels and then transferred onto nitrocellulose membranes. After blocked with 5% BSA for 1 h at room temperature, the membranes were incubated with primary antibodies at 4°C overnight and then with secondary antibodies at room temperature for 2 h. The protein bands were luminesced using Pierce^TM^ ECL Western Blotting Substrate, detected and analyzed with Bio-Rad ChemiDoc sytem. GAPDH or β-actin were used as endogenous control, and TATA box binding protein (TBP) was used as the endogenous control of nuclear protein. Antibodies used in this study were as followed: NR4A1 (abcam, 1:500), p65 (Cell Signaling Technology, 1:1000); GAPDH (Cell Signaling Technology, 1:2000), β-actin (Cell Signaling Technology, 1:2000), Cox-2 (abcam, 1:1000), iNOS (R&D, 1:1000), MMP3 (Cell Signaling Technology, 1:1000), MMP9 (abcam, 1:1000), MMP13 (Cell Signaling Technology, 1:1000), p-NR4A1 (Cell Signaling Technology, 1:1000), acetyl H4 lys8 (Cell Signaling Technology, 1:600), acetyl H3 lys27 (Cell Signaling Technology, 1:600), p-p65 (Cell Signaling Technology, 1:1000), IKBa (Cell Signaling Technology, 1:1000), p38 (Cell Signaling Technology, 1:1000), p-p38 (Cell Signaling Technology, 1:1000), ERK (Cell Signaling Technology, 1:1000), p-ERK (Cell Signaling Technology, 1:1000), JNK (Cell Signaling Technology, 1:1000), p-JNK (Cell Signaling Technology, 1:1000), TBP (Cell Signaling Technology, 1:1000). Second antibodies were purchased from Beyotime Biotechnology (China) and incubated with a concentration of 1:2000.

### NR4A1 Overexpression and siRNA

Rat NR4A1 overexpression plasmid and control plasmid were purchased from Haogene Company (Hangzhou, China). NR4A1 siRNA were purchased from Genepharma Technology Co., Ltd. (Shanghai, China). The transfection of plasmid and siRNA were conducted by Lipofectamine 3000 (Thermo Fisher Scientific) according to the manufacturer’s protocol. 24 h after transfection, the medium was changed with serum free medium and IL-1β were co-incubated for another 24 h before further testing.

### Luciferase Reporter Gene Assay

The pGL4.32 [luc2P/NF-κB-RE/Hygro] Vector, control vector pGL4.75[hRluc/CMV] Vector and Dual-Luciferase^®^ Reporter Assay System were purchased from Promega Biotech Co., Ltd., Rat chondrocytes were plated in 96-well plates and allowed to grow to approximately 75% confluency. Then the luciferase reporter vectors were co-transfected with NR4A1 overexpression vector or siRNA with Lipofectamine 3000. 24 h after transfection, the medium was changed as serum free medium, and IL-1β with a final concentration of 10 ng/ml or equal volume of PBS was added. After 5 h incubation, the luciferase activities were detected with Dual-Luciferase^®^ Reporter Assay System according to the manufacturer’s protocol. The relative luciferase activity was calculated as firefly luciferase activity/renilla luciferase activity and was normalized to negative control group.

### Animal Experiments and Histological Analysis

Five sham surgeries and ten OA models were developed by simply opening joint cavity or surgical resection of the medial meniscus in SD rat (Male, 6 weeks old, specific pathogen free [SPF])knee joints as described previously (Ran et al., 2018). All the OA model animals were randomized into two groups: the experimental group (*n* = 5) and the control group (*n* = 5). One week after surgery, 50 μl of 1 μM cytosporone B (Experimental group) or equal volume of vehicle (Control group) were injected intra-articularly every week. Six weeks post operation, the animals were sacrificed and the knee samples were collected for safranin O (SO) staining. OARSI scores (Pritzker et al., 2006), which is commonly used in assessing cartilage destruction histology and the higher grades indicate more severe cartilage damage, were graded by two independent researchers to evaluate the OA severity. The animal experiments were conducted in accordance with NIH guidelines (NIH Pub. No. 85-23, revised 1996), and the protocol was approved by the Ethics Committee of The Second Affiliated Hospital, School of Medicine, Zhejiang University, Hangzhou, China.

### Immunofluorescence and Immunohistochemistry

For paraffin embedded tissue samples, histological sections were prepared, deparaffinized and hydrated gradiently. For immunohistochemistry, the hydrated sections were firstly undergone heat antigen retrieval and then blocked with hydrogen peroxide for 20 min at room temperature. Thereafter, the sections were blocked with 5% BSA for 1 h at room temperature and incubated with primary antibody (NR4A1, Cell Signaling Technology, 1:100) or IgG control (Cell Signaling Technology, 1:100) over night at 4°C. Then the sections were incubated with HRP-linked secondary antibodies (Beyotime Biotechnology, 1:500) for 1 h at room temperature and 3,30-diaminobenzidine was used as a chromogenic agent. For immunofluorescence, the hydrogen peroxide blocking procedure was not conducted. After primary antibodies (NR4A1, Cell Signaling Technology, 1:100; p65, Cell Signaling Technology, 1:100) or IgG control (Cell Signaling Technology, 1:100) incubation, the sections were incubated with FITC or Cy3 linked second antibodies (Beyotime Biotechnology, 1:500) for 1 h and then stained with DAPI for 5 min before observation under the fluorescence microscope.

### Statistical Analysis

All quantitative data sets are presented as mean ± SD. Student’s *t*-test was performed to assess statistically significant differences when comparing two groups, and 1-way ANOVA followed by Bonferroni’s *post hoc* test was used when comparing more than two groups. Statistical differences were performed with SPSS 20.0 version and values of *p* < 0.05 were considered to be significantly different.

## Results

### NR4A1 Was Up-Regulated in Osteoarthritis Tissue Through NF-κB Signal Pathway Activation

**FIGURE 1:**
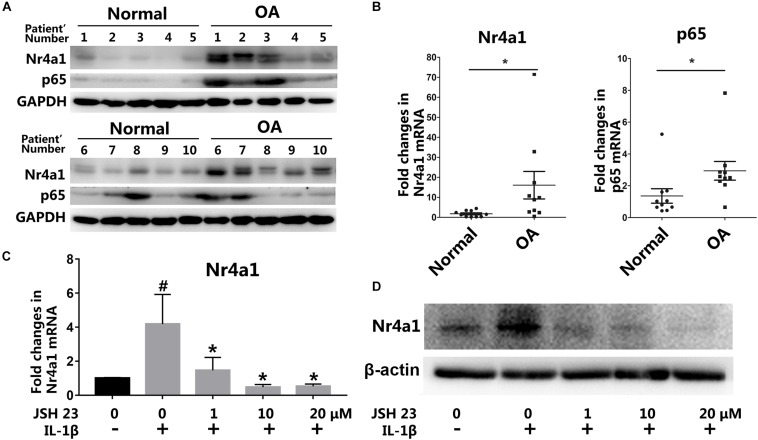
The expression of NR4A1 is induced in osteoarthritis. **(A,B)** The detection of NR4A1 and p65 expression in human osteoarthritis cartilage and normal cartilage through western-blot **(A)** and RT-PCR analysis **(B)**. **(C,D)** The effect of NF-κB signal inhibitor JSH23 on IL-1β induced NR4A1 expression in rat chondrocytes: **(C)** RT-PCR analysis; **(D)** Western-blot analysis. ^#^*p* < 0.05, compared with the negative control group. *p < 0.05, compared with the IL-1β group.

To investigate the function and mechanism of NR4A1 in osteoarthritis, we firstly detected the expression of NR4A1 and p65 in normal and OA cartilage by western blot and RT-PCR. The results showed that NR4A1 expression was up-regulated and correlated with p65 expression in human OA cartilage (Figures 1A,B and Supplementary Figure S1). This indicated that the up-regulation of NR4A1 may associate with NF-κB signal pathway activation. IL-1β treatment was demonstrated to increase the expression of NR4A1 significantly within 24 h in rat chondrocytes *in vitro*, which could be reversed by NF-κB pathway antagonist JSH-23 with a concentration dependent manner (Figures 1C,D). These data revealed that the expression of NR4A1 was up-regulated through the activation of NF-κB signal pathway in osteoarthritis.

### NR4A1 Inhibited Chondrocytes Inflammation and Matrix Metalloproteinases (MMPs) Expression via Suppression of NF-κB Signal Pathway

**FIGURE 2:**
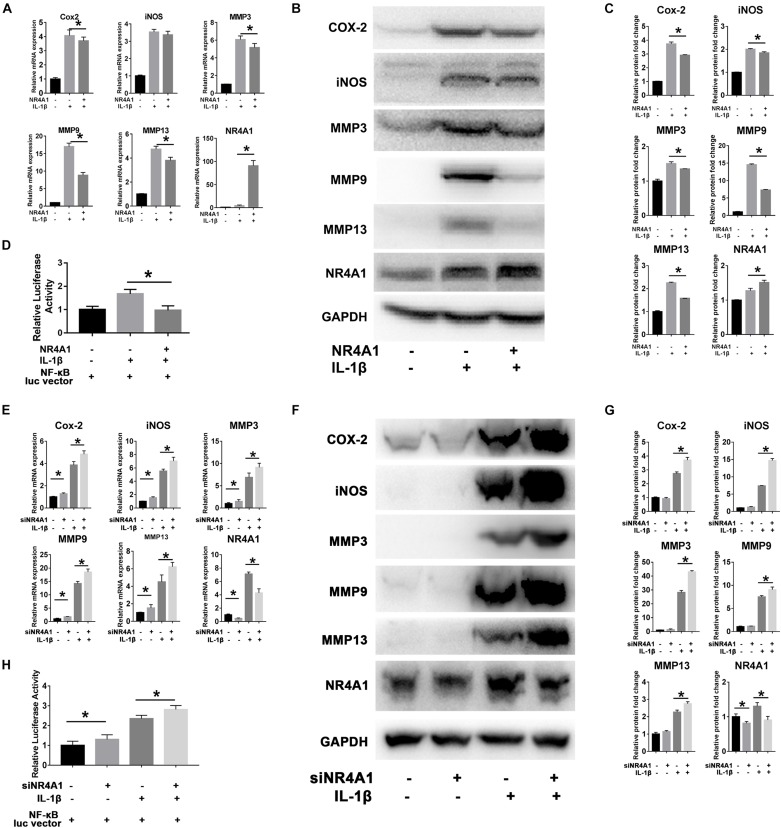
NR4A1 could inhibit NF-κB signal and chondrocyte inflammation associated gene expression. **(A–D)** The overexpression experiment: The RT-PCR analysis **(A)** and western-blot analysis **(B)** of the effect of NR4A1 overexpression on IL-1β induced COX-2, iNOS, MMP3, MMP9, MMP13 expression in rat chondrocytes. **(C)** Quantity analysis of the protein bands in panel **(B)**. **(D)** Luciferase reporter gene analysis to detect the transcriptional activity of NF-κB pathway. **(E–H)** The siRNA experiment: The RT-PCR analysis **(E)** and western-blot analysis **(F)** of the effect of NR4A1 knockdown on IL-1β induced COX-2, iNOS, MMP3, MMP9, MMP13 expression in rat chondrocytes. **(G)** Quantity analysis of the protein bands in panel **(F)**. **(H)** Luciferase reporter gene analysis to detect the transcriptional activity of NF-κB pathway. *p < 0.05.

Overexpression of NR4A1 could effectively inhibit IL-1β induced up-regulation of COX-2, MMP3, MMP9 and MMP13 in both protein and mRNA level in rat chondrocytes (Figures 2A–C). The luciferase reporter gene assay was then used to confirm the suppressive effect of NR4A1 on NF-κB pathway. The result showed that the relative luciferase activity driven by NF-κB response element in NR4A1 overexpression group was significantly lower than the IL-1β stimulated group (Figure 2D).

On the contrary, the knockdown of NR4A1 by siRNA transfection before IL-1β treatment resulted in higher expression of COX-2, iNOS, MMP3, MMP9, and MMP13 genes than IL-1β treatment alone in rat chondrocytes (Figures 2E–G). The relative luciferase activity driven by NF-κB response element was significantly elevated in the NR4A1 knockdown group (Figure 2H).

These results demonstrated NR4A1 as a natural negative feedback regulator of NF-κB signal pathway and could suppress chondrocyte inflammation associated genes expression.

### NR4A1 Was Relatively Inactivated in Osteoarthritis Through HDACs Mediated Transcriptional Suppression and MAPK Dependent Phosphorylation

**FIGURE 3:**
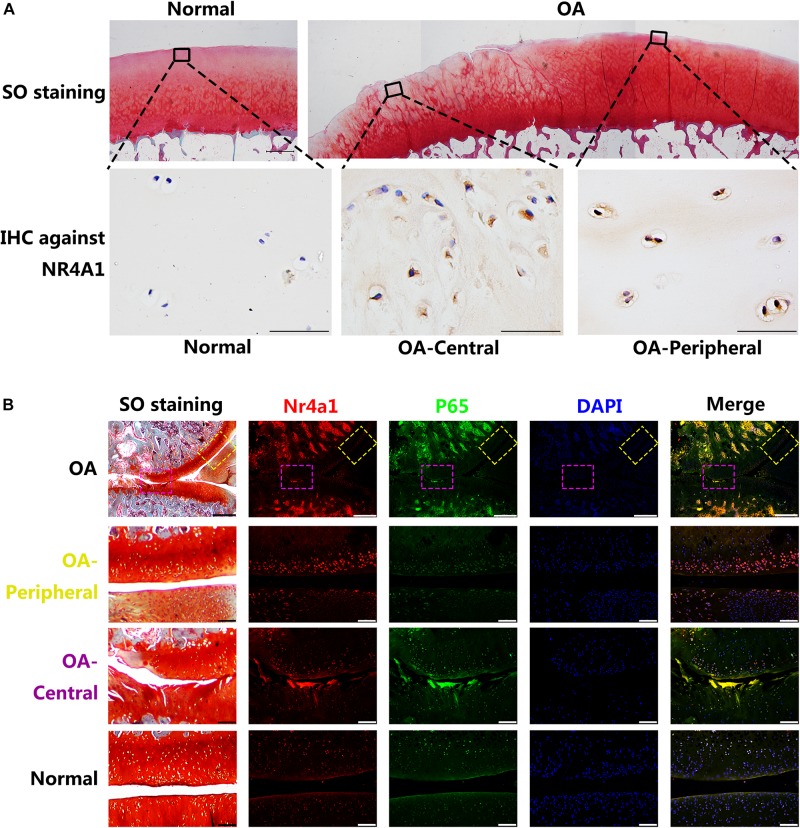
The NR4A1 is relatively inhibited in the central area of OA. **(A)** Human normal and OA samples. The upper row: SO staining, scale bar = 1 mm. The Lower row: Immunohistochemistry (IHC) against NR4A1, scale bar = 50 μm. **(B)** Rat OA models. Yellow dotted box: relatively normal area in OA joint. Purple dotted box: the central area of OA joint. Scale bar = 100 μm.

The intensity and distribution of the expression of NR4A1 in rat OA model and human OA cartilage samples were further investigated. The immunohistochemistry revealed that the NR4A1 positive rate was increased in human OA cartilage and it’s interesting that the positive rate in the central area of erosion was lower than that in the peripheral area where the cartilage structure remained relatively normal (Figure 3A). Similar results were observed in rat OA model samples by the immunofluorescence analysis. In the central area of rat OA cartilage, the expression of p65 was highly elevated and the expression of NR4A1 was moderately increased. However, in the peripheral area of rat OA cartilage, where normal cartilage structure remained, the expression of p65 was moderately elevated whereas the expression of NR4A1 was highly increased (Figure 3B). These results supposed that NR4A1 signaling might be impaired in osteoarthritis and persistently inflammatory stimulation could result in the inactivation of NR4A1 response.

**FIGURE 4:**
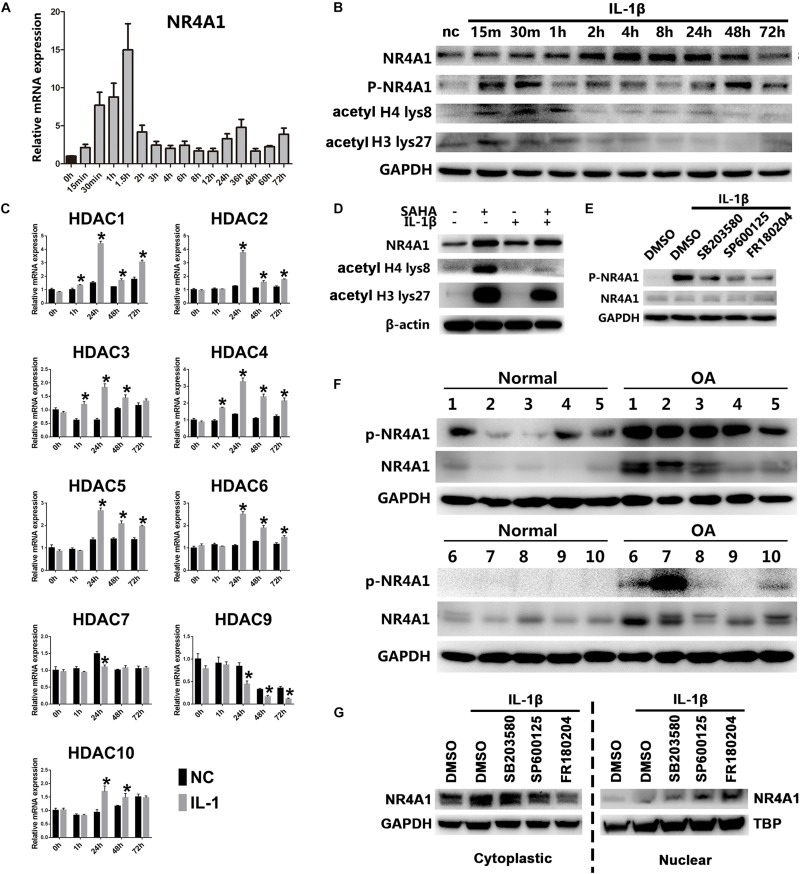
NR4A1 was relatively inactivated in osteoarthritis through HDACs mediated transcriptional suppression and MAKP dependent phosphorylation. **(A,B)** The expression of NR4A1 in conditions of chronic NF-κB signal activation. **(A)** The mRNA level. **(B)** The protein level of NR4A1, phosphorylated NR4A1, acetyl H4 lys8 and acetyl H3 lys27. Chondrocytes treated with 10 ng/ml IL-1β for different time were collected for the detection. nc, negative control. **(C)** The dynamic expression of HDACs under chronic NF-κB signal activation; *p < 0.05 compared with negative control at the same time point. **(D)** The effect of HDAC inhibitor SAHA (5 μM) on the NR4A1 expression, acetyl H4 lys8 and acetyl H3 lys27 after 72 h IL-1β treatment. **(E)** The effect of p38 inhibitor SB203580, JNK inhibitor SP600125 and ERK inhibitor FR180204 on NR4A1 phosphorylation. **(F)** The NR4A1 and phosphorylated NR4A1 level in human osteoarthritis and normal cartilage tissues. **(G)** The effect of p38 inhibitor SB203580, JNK inhibitor SP600125 and ERK inhibitor FR180204 on NR4A1 subcellular location.

Thus, we exposed rat chondrocytes to chronic IL-1β for prolonged periods *in vitro* and the results showed that the mRNA and protein level of NR4A1 declined rapidly after an initial peak (Figures 4A,B). Since histone acetylation may participate in regulation NR4A1 expression (Palumbo-Zerr et al., 2015), we detected the acetylated level at histone 4 (H4) lys8 and histone 3 (H3) lys27 in rat chondrocytes under chronic IL-1β stimulation by western-blot. The results showed that the acetylated level in both H4 lys8 and H3 lys27 increased dramatically after IL-1β treatment and dropped rapidly ever since (Figure 4B). We further investigated the dynamic mRNA expression of representative histone deacetylases (HDACs) and found that HDAC 1,2,3,4,5,6,10 were upregulated under long-term IL-1β treatment (Figure 4C). The pan-HDAC inhibitor SAHA could prevent the lower degree of NR4A1 expression under chronic stimulation of IL-1β (Figure 4D).

The phosphorylation of NR4A1 is another important regulatory mechanism that reduces the transcriptional activity and inhibitory ability against NF-κB signal (Li et al., 2015). We detected that NR4A1 was highly phosphorylated in human OA cartilage tissue (Figure 4F). NR4A1 phosphorylation could be induced by IL-1β treatment *in vitro* (Figures 4B,E), which was significantly inhibited by p38 inhibitor SB203580, JNK inhibitor SP600125 and ERK inhibitor FR180204 (Figure 4E). Moreover, the cytoplasmic NR4A1 increased under IL-1β stimulation, whereas JNK inhibitor SP600125 and ERK inhibitor FR180204 decreased the cytoplasmic NR4A1 and increased nuclear NR4A1 (Figure 4G).

These results suggested that chronic IL-1β stimulation in osteoarthritis inactivates the physiologic NR4A1 negative feed-back loop by HDAC-mediated epigenetic silencing and MAPK- dependent phosphorylation.

### Reactivation of NR4A1 by Cytosporone B Attenuates Chondrocyte Inflammation and OA Development *in vitro* and *in vivo*

**FIGURE 5:**
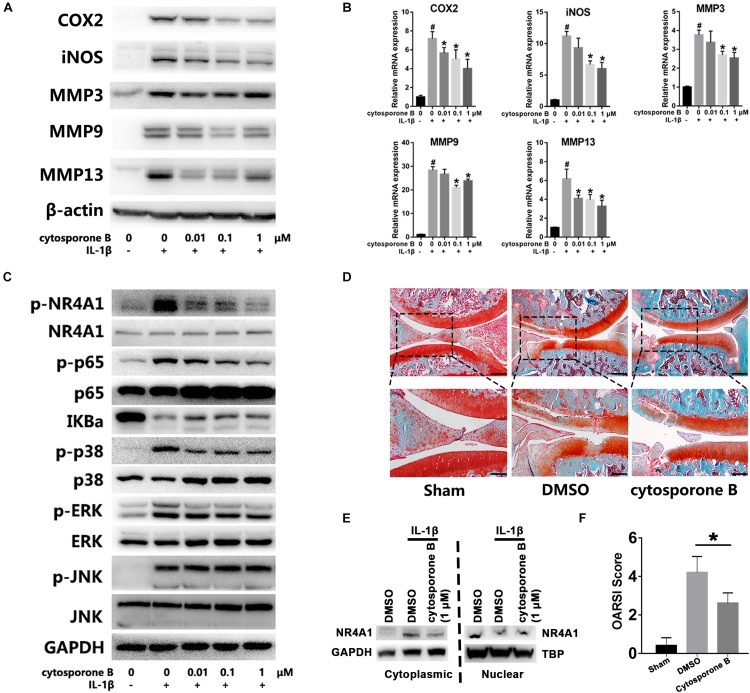
Cytosporone B reactivated NR4A1 and attenuated chondrocyte inflammation and OA development *in vitro* and *in vivo*. **(A,B)** The effect of cytosporone B on IL-1β induced COX2, iNOS, MMP3, MMP9 and MMP13 expression. **(A)** Protein level. **(B)** mRNA level. **(C)** The effect of cytosporone B on NR4A1 phosphorylation, NF-κB signal activation and MAPK pathway activation. **(D)** SO staining of sham surgery and rat knee joint OA model treated with 1 μM cytosporone B or vehicle. Scale bar = 500 μm (upper), 250 μm (lower). **(E)** The effect of 1 μM cytosporone B on NR4A1 subcellular location. **(F)** OARSI scores of each group. *p < 0.05.

At last, we accessed the effect and mechanism of NR4A1 agonist cytosporone B on chondrocytes inflammation and OA development. The results showed that cytosporone B could significantly inhibit IL-1β induced COX-2, iNOS, MMP3, MMP9 and MMP13 expression in a concentration dependent manner (Figures 5A,B). Further investigation revealed that cytosporone B significantly reduced the IL-1β induced phosphorylation of NR4A1 through the inhibition of p38, JNK and ERK phosphorylation. The phosphorylation of p65 and degradation of IKBa were also partially restrained by cytosporone B (Figure 5C). We also found that 1 μM cytosporone B could significantly decrease the cytoplasmic NR4A1 and increase the nuclear NR4A1 (Figure 5E). In the rat OA model, the SO staining demonstrated that the articular injection of cytosporone B exhibited decreased cartilage destruction, attenuated OA severity and significantly lower OARSI score (*p* < 0.05) (Figures 5D,F).

## Discussion

**FIGURE 6:**
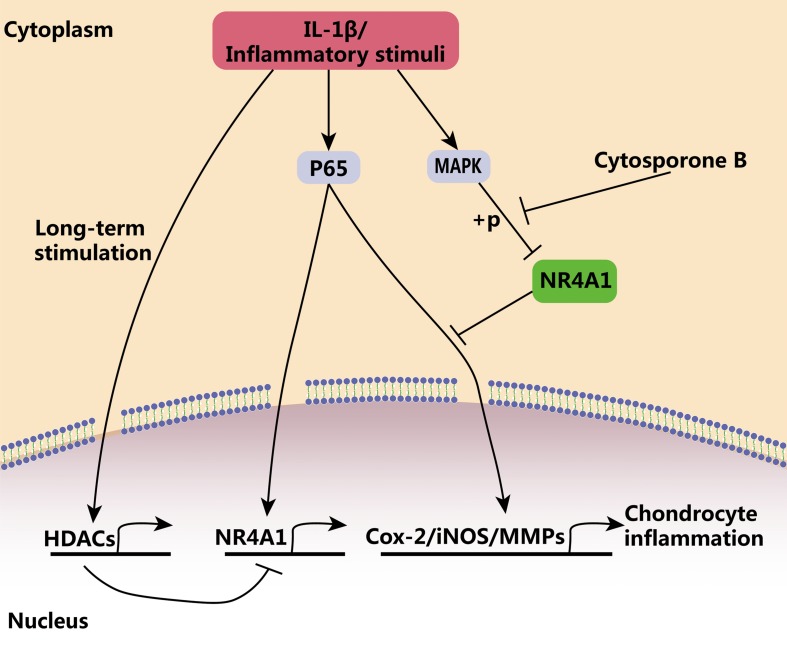
The schematic diagram of the regulatory role of NR4A1 in chondrocyte inflammation and osteoarthritis. NR4A1 acts as an endogenous inhibitor of p65 and suppresses chondrocyte inflammation. However, NR4A1 was relatively inactivated in osteoarthritis through HDACs mediated transcriptional suppression and MAPK dependent phosphorylation. Cytosporone B can reactivate NR4A1 and attenuate chondrocyte inflammation.

In the present study, we demonstrated that NR4A1 was a key checkpoint for inflammatory response and the failure of its negative regulatory ability existed in advanced osteoarthritis. NF-κB signaling, a central pathway of inflammatory response, plays vital roles in immune defense and tissue injury repair (Chen and Nunez, 2010; Rathinam and Chan, 2018). However, the persistent activation of inflammatory response and NF-κB signaling always results in tissue dysfunction and destruction (Afonina et al., 2017; Zhang et al., 2017). Our results demonstrated that transit NF-κB signal activation induced NR4A1 expression, which in turn blocks the uncontrolled and prolonged activation of NF-κB signal and prevent chondrocyte inflammation. However, persistent NF-κB activation relatively inhibited NR4A1 expression through HDAC mediated epigenetic silencing. On the other hand, the activation of NF-κB phosphorylated NR4A1 in a MAPK dependent mechanism and weakened the inhibitory effect of NR41. Thus, NR4A1 was relatively inactivated in osteoarthritis and thereby the NF-κB signal got out of the physiological negative regulation by NR4A1. The reactivation of NR4A1 by its agonist cytosporone B could prevent chondrocyte inflammation and ameliorate osteoarthritis *in vitro* and *in vivo* (Figure 6).

As a rapid response regulator, the NR4A1 has a low baseline expression level and can be rapidly and highly induced by a variety of stimulations including inflammation. NR4A3, another member of the NR4A subfamilies, has been proved to plays a pro-inflammatory role in the development of OA via the NF-κB pathway (Ma et al., 2020). However, the role and mechanism of NR4A1 in chondrocyte inflammation and OA progress was not fully investigated. Through a genome-wide survey, Diatchenko L et al. demonstrated that NR4A1 is one of the most effective endogenous inhibitor of NF-κB signal in monocytes (Diatchenko et al., 2005). In inflammation-associated diseases like coronary atherosclerosis (You et al., 2009; Bonta et al., 2010), tumor (Lee et al., 2011; To et al., 2012) and neurodegenerative disease (Montarolo et al., 2016; Wei et al., 2016), NR4A1 was proved to be up-regulated and approaches targeting NR4A1 could partially ameliorate disease progression. In the present study, our data revealed that the expression of NR4A1 was also elevated in osteoarthritis cartilage tissue through the activation of NF-κB signal pathway. *In vitro* overexpression and knockdown experiments demonstrated that NR4A1 could inhibit the p65 transcriptional ability and prevent the IL-1β induced MMPs expression in chondrocytes, thus indicating a protective role of NR4A1 in osteoarthritis. Interestingly, the upregulation of NR4A1 expression *in situ* was out of expectation that the NR4A1 was relatively suppressed in the central area of OA cartilage where the tissue damage was more severe. This phenomenon enlightened that the regulation of NR4A1 expression and activation was complex in OA.

Unlike other nuclear receptors, whose structure typically consists a C-terminal ligand-binding domain (LBD), a central DNA binding domain, and an N-terminal activation function 1 domain (Mangelsdorf et al., 1995), the LBD of NR4A1 is atypical and thought to be unable to directly mediate cofactor recruitment (Wansa et al., 2002). Therefore, the regulation of NR4A1 mainly includes transcriptional regulation and phosphorylation regulation. Katrin Palumbo-Zerr et al. firstly reported the distinct effects of transit and persistent TGF-β signal stimulation on NR4A1 where transit stimulation promoted NR4A1 expression and long-term stimulation inhibited NR4A1 expression and activation (Palumbo-Zerr et al., 2015). Our result confirmed a similar regulation pattern that transit inflammatory stimulation significantly induce NR4A1 expression through the activation of NF-κB signal whereas the long-term stimulation also promoted HDACs expression and thus inhibited the NR4A1 expression. Our results were consistent with previous studies that HDAC inhibitors could restore the expression of silenced NR4A1 in pathological condition (Zhou et al., 2013; Palumbo-Zerr et al., 2015). The phosphorylation of NR4A1, which leads to the inactivation and cytoplasmic translocation of NR4A1, is another important regulation mechanism. The MAPK kinase p38, ERK and JNK were demonstrated to phosphorylate NR4A1 in other cell lines (Liu et al., 2007; Wang et al., 2009; Li et al., 2015). Our previous study has revealed that the inflammatory stimulation phosphorylated and activated MAPK p38, ERK and JNK in chondrocytes (Ran et al., 2018). In the present study, we observed that NR4A1 was phosphorylated in osteoarthritis tissue or through *in vitro* inflammatory stimulation, which was significantly blocked by p38, ERK and JNK inhibitors. Thus inflammation could phosphorylate and inactivate NR4A1 in osteoarthritis through the activation of MAPK pathway.

Despite the anti-inflammatory function, NR4A1 was also recognized to be able to bind to mitochondria and promote apoptosis and death (Wenzl et al., 2015; Herring et al., 2019). The opposite effects on cell inflammation and survival reminded us that NR4A1 was subtly regulated and may play different roles in different cell types or the same cell type but under different circumstance. Shi et al. (2017) found that the upregulated NR4A1 in OA was mostly located in mitochondria and knockdown of NR4A1 could decrease the cleaved PARP1 expression and avoid apoptosis of OA chondrocytes. Zheng et al. (2020) also revealed that NR4A1 promotes TNF-α-induced chondrocyte death via activating mitochondrial fission. These reports seemed to indicate that NR4A1 acted as a harmful factor in OA. However, previous studies demonstrated that the phosphorylation state is a crucial factor that regulating NR4A1 location and function (Wang et al., 2009; Li et al., 2015). Phosphorylation of NR4A1 will lead to its translocation from the nuclear to the mitochondria and promoting apoptosis (Wang et al., 2009; Hedrick and Safe, 2017; Hedrick et al., 2018), and also abolish the suppressive function on NF-κB pathway activation (Li et al., 2015). Our study demonstrated that NR4A1 was highly phosphorylated in OA chondrocytes, which could explain the mitochondria translocation observed in Xinge’s research (Shi et al., 2017). Thus, it’s the phosphorylation and inactivating of NR4A1, rather than the protein expression, that lead to chondrocyte apoptosis and death. Our study also demonstrated that dephosphorylation and reactivation of NR4A1 could inhibit its cytoplasmic accumulation and restore the anti-inflammation ability without incensement of apoptosis.

Up to now, the endogenous ligand of NR4A1 was still not identified. Several small molecules have been proved to directly regulate NR4A1 activity. Cytosporone B (Zhan et al., 2008) and ethyl [2,3,4-trimethoxy-6-(i-octanoyl) phenyl] acetate (TMPA) (Zhan et al., 2012) could activate whereas 1-(3,4,5-trihydroxyphenyl) nonan-1-one (THPN) (Wang et al., 2014) and bis-indole derived compounds (Hedrick et al., 2019; Karki et al., 2020) could inhibit NR4A1. In our study, we confirmed that cytosporone B could reactivate NR4A1 under inflammatory stimulation through the inhibition of NR4A1 phosphorylation, which enhanced the endogenous inhibitory regulatory loop of NF-κB signal and reduce the MMPs expression and chondrocytes inflammation. The mechanism that cytosporone B regulates NR4A1 depends on a direct contact to the ligand binding domain, which was close to the phosphorylation residues in the crystal structure (Zhan et al., 2008), thus may also influence its phosphorylation. Besides, MAPKs showed abilities to phosphorylate NR4A1 (Figure 4E) and cytosporone B could also decreased p38 phosphorylation without apparent influences on JNK and ERK phosphorylation (Figure 5C), so cytosporone B may also inhibit NR4A1 phosphorylation thorough a p38 dependent manner. Though the NR4A1 agonist showed therapeutic effects of OA *in vivo* and *in vitro*, the potential side effects should be considered. High dose cytosporone B (over 10 μM) induced mitochondria translocation and subsequent apoptosis and exhibited anti-tumor effects in tumor cells (Cho et al., 2007; Zhan et al., 2008), however, clinically relevant concentration of cytosporone B did not increase apoptosis in non-tumor cells (Palumbo-Zerr et al., 2015). In our study, 1 μM cytosporone B treatment didn’t promote NR4A1 cytoplasmic translocation but inhibited the IL-1β induced phosphorylation and cytoplasmic accumulation in chondrocytes. The different effects on NR4A1 subcellular locations may because of the different concentrations of cytosporone B and the different cell types, however, the exact mechanism remains unclear. The therapeutic concentration of cytosporone B (0.1–1 μM) in our study was over 10 fold lower than the anti-tumor concentration and chondrocytes apoptosis was not obviously observed under 1 μM cytosporone B treatment.

Though our study gave promising results, there’re some limitations. Because of the lack of human heathy chondrocytes, the cells used in our study are mainly rat chondrocytes, which may not to fully mimic the characteristics of human cartilage. Since there’s currently no consensus model for OA, we developed the OA model by surgical resection of medial meniscus in rats, which was the most commonly used in OA researches. However, this OA model may not naturally reflect human disease. Further studies in large animal model regarding the effectiveness and safety of NR4A1 activation in treating OA are needed, which may help to promote the potential clinic translation.

In conclusion, we demonstrated that the NR4A1 is a key endogenous inhibitor of chondrocyte inflammation, which was relatively inactivated under chronic inflammatory stimulation through HDACs mediated transcriptional suppression and MAKP dependent phosphorylation in osteoarthritis. NR4A1 agonist cytosporone B could reactivate and restore the inhibitory regulatory ability of NR4A1, prevent excessive inflammation, and ameliorates osteoarthritis.

## Data Availability Statement

All datasets generated for this study are included in the article/Supplementary Material.

## Ethics Statement

The studies involving human participants were reviewed and approved by the Ethics Committee of The Second Affiliated Hospital, School of Medicine, Zhejiang University, Hangzhou, China. The patients/participants provided their written informed consent to participate in this study. The animal study was reviewed and approved by the Ethics Committee of The Second Affiliated Hospital, School of Medicine, Zhejiang University, Hangzhou, China.

## Author Contributions

LW conceived and designed the study. JR, LX, ZT, CM, KX, YH, MA, PH, WC, and ZC performed the *in vitro* analysis, animal experiments, interpreted the data, and conducted the statistical analysis. YX and JR wrote the manuscript. All the authors finally approved the manuscript.

## Conflict of Interest

The authors declare that the research was conducted in the absence of any commercial or financial relationships that could be construed as a potential conflict of interest.

## Supplementary Material

FIGURE S1 The correlation analysis of p65 expression and NR4A1 expression in human samples. **(A)** Total patients. **(B)** Normal group. **(C)** OA group.

TABLE S1 Primer sequences used in this study.

## References

[B1] Afonina I. S. Zhong Z. Karin M. Beyaert R. (2017). Limiting inflammation-the negative regulation of NF-kappaB and the NLRP3 inflammasome. *Nat. Immunol.* 18 861–869. 10.1038/ni.3772 28722711

[B2] Alessandri A. L. Sousa L. P. Lucas C. D. Rossi A. G. Pinho V. Teixeira M. M. (2013). Resolution of inflammation: mechanisms and opportunity for drug development. *Pharmacol. Therapeut.* 139 189–212. 10.1016/j.pharmthera.2013.04.006 23583354

[B3] Berenbaum F. Griffin T. M. Liu-Bryan R. (2017). Review: metabolic regulation of inflammation in osteoarthritis. *Arthrit. Rheumatol.* 69 9–21. 10.1002/art.39842 27564539 PMC5341385

[B4] Bonta P. I. Matlung H. L. Vos M. Peters S. L. Pannekoek H. Bakker E. N. (2010). Nuclear receptor Nur77 inhibits vascular outward remodelling and reduces macrophage accumulation and matrix metalloproteinase levels. *Cardiovasc. Res.* 87 561–568. 10.1093/cvr/cvq064 20189954

[B5] Chen G. Y. Nunez G. (2010). Sterile inflammation: sensing and reacting to damage. *Nat. Rev. Immunol.* 10 826–837. 10.1038/nri2873 21088683 PMC3114424

[B6] Cho S. D. Yoon K. Chintharlapalli S. Abdelrahim M. Lei P. Hamilton S. (2007). Nur77 agonists induce proapoptotic genes and responses in colon cancer cells through nuclear receptor-dependent and nuclear receptor-independent pathways. *Cancer Res.* 67 674–683. 10.1158/0008-5472.can-06-2907 17234778

[B7] Diatchenko L. Romanov S. Malinina I. Clarke J. Tchivilev I. Li X. (2005). Identification of novel mediators of NF-kappaB through genome-wide survey of monocyte adherence-induced genes. *J. Leukocyte Biol.* 78 1366–1377. 10.1189/jlb.0405211 16204640

[B8] Gilroy D. De Maeyer R. (2015). New insights into the resolution of inflammation. *Semin. Immunol.* 27 161–168. 10.1016/j.smim.2015.05.003 26037968

[B9] Goldring S. R. Goldring M. B. (2016). Changes in the osteochondral unit during osteoarthritis: structure, function and cartilage-bone crosstalk. *Nat. Rev. Rheumatol.* 12 632–644. 10.1038/nrrheum.2016.148 27652499

[B10] Hedrick E. Li X. Cheng Y. Lacey A. Mohankumar K. Zarei M. (2019). Potent inhibition of breast cancer by bis-indole-derived nuclear receptor 4A1 (NR4A1) antagonists. *Breast Cancer Res. Treat.* 177 29–40. 10.1007/s10549-019-05279-9 31119568 PMC6681651

[B11] Hedrick E. Mohankumar K. Safe S. (2018). TGFbeta-Induced Lung Cancer Cell Migration Is NR4A1-Dependent. *Mol. Cancer Res.* 16 1991–2002. 10.1158/1541-7786.MCR-18-0366 30072581 PMC6343492

[B12] Hedrick E. Safe S. (2017). Transforming growth factor beta/NR4A1-inducible breast cancer cell migration and epithelial-to-Mesenchymal transition is p38alpha (Mitogen-activated protein Kinase 14) dependent. *Mol. Cell. Biol.* 37 e306–e317. 10.1128/MCB.00306-17 28674186 PMC5574050

[B13] Herring J. A. Elison W. S. Tessem J. S. (2019). Function of Nr4a orphan nuclear receptors in proliferation, apoptosis and fuel utilization across tissues. *Cells* 8:1373. 10.3390/cells8111373 31683815 PMC6912296

[B14] Karki K. Wright G. A. Mohankumar K. Jin U. H. Zhang X. H. Safe S. (2020). A Bis-Indole-Derived NR4A1 antagonist induces PD-L1 degradation 1248 and enhances anti-tumor immunity. *Cancer Res.* 80 1011–1023. 10.1158/0008-5472.CAN-19-2314 31911554 PMC7056589

[B15] Lee S. O. Li X. Khan S. Safe S. (2011). Targeting NR4A1 (TR3) in cancer cells and tumors. *Expert. Opin. Ther. Targets* 15 195–206. 10.1517/14728222.2011.547481 21204731 PMC4407471

[B16] Li L. Liu Y. Chen H. Z. Li F. W. Wu J. F. Zhang H. K. (2015). Impeding the interaction between Nur77 and p38 reduces LPS-induced inflammation. *Nat. Chem. Biol.* 11 339–346. 10.1038/nchembio.1788 25822914

[B17] Liu B. Wu J. F. Zhan Y. Y. Chen H. Z. Zhang X. Y. Wu Q. (2007). Regulation of the orphan receptor TR3 nuclear functions by c-Jun N terminal kinase phosphorylation. *Endocrinology* 148 34–44. 10.1210/en.2006-0800 17023523

[B18] Liu-Bryan R. Terkeltaub R. (2015). Emerging regulators of the inflammatory process in osteoarthritis. *Nat. Rev. Rheumatol.* 11 35–44. 10.1038/nrrheum.2014.162 25266449 PMC4374654

[B19] Ma C. Wu L. Song L. He Y. Adel Abdo Moqbel S. Yan S. (2020). The pro-inflammatory effect of NR4A3 in osteoarthritis. *J. Cell. Mol. Med.* 24 930–940. 10.1111/jcmm.14804 31701670 PMC6933326

[B20] Mangelsdorf D. J. Thummel C. Beato M. Herrlich P. Schutz G. Umesono K. (1995). The nuclear receptor superfamily: the second decade. *Cell* 83 835–839. 10.1016/0092-8674(95)90199-x 8521507 PMC6159888

[B21] Medzhitov R. (2008). Origin and physiological roles of inflammation. *Nature* 454 428–435. 10.1038/nature07201 18650913

[B22] Montarolo F. Perga S. Martire S. Navone D. N. Marchet A. Leotta D. (2016). Altered NR4A subfamily gene expression level in peripheral blood of parkinson’s and alzheimer’s disease patients. *Neurotox. Res.* 30 338–344. 10.1007/s12640-016-9626-4 27159982

[B23] Palumbo-Zerr K. Zerr P. Distler A. Fliehr J. Mancuso R. Huang J. (2015). Orphan nuclear receptor NR4A1 regulates transforming growth factor-beta signaling and fibrosis. *Nat. Med.* 21 150–158. 10.1038/nm.3777 25581517

[B24] Pei L. Castrillo A. Chen M. Hoffmann A. Tontonoz P. (2005). Induction of NR4A orphan nuclear receptor expression in macrophages in response to inflammatory stimuli. *J. Biol. Chem.* 280 29256–29262. 10.1074/jbc.m502606200 15964844

[B25] Pritzker K. P. Gay S. Jimenez S. A. Ostergaard K. Pelletier J. P. Revell P. A. (2006). Osteoarthritis cartilage histopathology: grading and staging. *Osteoarthr. Cartil.* 14 13–29. 10.1016/j.joca.2005.07.014 16242352

[B26] Ran J. Ma C. Xu K. Xu L. He Y. Moqbel S. A. A. (2018). Schisandrin B ameliorated chondrocytes inflammation and osteoarthritis via suppression of NF-kappaB and MAPK signal pathways. *Drug Design Dev. Ther.* 12 1195–1204. 10.2147/DDDT.S162014 29785089 PMC5953308

[B27] Rathinam V. A. K. Chan F. K. (2018). Inflammasome, Inflammation, and Tissue Homeostasis. *Trends Mol. Med.* 24 304–318. 10.1016/j.molmed.2018.01.004 29433944 PMC6456255

[B28] Robinson W. H. Lepus C. M. Wang Q. Raghu H. Mao R. Lindstrom T. M. (2016). Low-grade inflammation as a key mediator of the pathogenesis of osteoarthritis. *Nat. Rev. Rheumatol.* 12 580–592. 10.1038/nrrheum.2016.136 27539668 PMC5500215

[B29] Rodriguez-Calvo R. Tajes M. Vazquez-Carrera M. (2017). The NR4A subfamily of nuclear receptors: potential new therapeutic targets for the treatment of inflammatory diseases. *Expert Opin. Therap. Targets* 21 291–304. 10.1080/14728222.2017.1279146 28055275

[B30] Roos E. M. Arden N. K. (2015). Strategies for the prevention of knee osteoarthritis. *Nat. Rev. Rheumatol.* 12 92–101. 26439406 10.1038/nrrheum.2015.135

[B31] Scanzello C. R. (2016). Chemokines and inflammation in osteoarthritis: Insights from patients and animal models. *J. Orthop. Res.* 35 735–739. 10.1002/jor.23471 27808445 PMC5912941

[B32] Shaked I. Hanna R. N. Shaked H. Chodaczek G. Nowyhed H. N. Tweet G. (2015). Transcription factor Nr4a1 couples sympathetic and inflammatory cues in CNS-recruited macrophages to limit neuroinflammation. *Nat. Immunol.* 16 1228–1234. 10.1038/ni.3321 26523867 PMC4833087

[B33] Shi X. Ye H. Yao X. Gao Y. (2017). The involvement and possible mechanism of NR4A1 in chondrocyte apoptosis during osteoarthritis. *Am. J. Transl. Res.* 9 746–754. 28337303 PMC5340710

[B34] Sugimoto M. A. Sousa L. P. Pinho V. Perretti M. Teixeira M. M. (2016). Resolution of inflammation: what controls its onset? *Front. Immunol.* 7:160. 10.3389/fimmu.2016.00160 27199985 PMC4845539

[B35] To S. K. Zeng J. Z. Wong A. S. (2012). Nur77: a potential therapeutic target in cancer. *Expert Opin. Ther. Targets* 16 573–585. 10.1517/14728222.2012.680958 22537097

[B36] Wang A. Rud J. Olson C. M. Jr. Anguita J. Osborne B. A. (2009). Phosphorylation of Nur77 by the MEK-ERK-RSK cascade induces mitochondrial translocation and apoptosis in T cells. *J. Immunol.* 183 3268–3277. 10.4049/jimmunol.0900894 19675165

[B37] Wang W. J. Wang Y. Chen H. Z. Xing Y. Z. Li F. W. Zhang Q. (2014). Orphan nuclear receptor TR3 acts in autophagic cell death via mitochondrial signaling pathway. *Nat. Chem. Biol.* 10 133–140. 10.1038/nchembio.1406 24316735

[B38] Wansa K. D. Harris J. M. Muscat G. E. (2002). The activation function-1 domain of Nur77/NR4A1 mediates trans-activation, cell specificity, and coactivator recruitment. *J. Biol. Chem.* 277 33001–33011. 10.1074/jbc.m203572200 12082103

[B39] Wei X. Gao H. Zou J. Liu X. Chen D. Liao J. (2016). Contra-directional coupling of Nur77 and Nurr1 in neurodegeneration: a novel mechanism for memantine-induced anti-inflammation and anti-mitochondrial impairment. *Mol. Neurobiol.* 53 5876–5892. 10.1007/s12035-015-9477-7 26497037

[B40] Wenzl K. Troppan K. Neumeister P. Deutsch A. J. (2015). The nuclear orphan receptor NR4A1 and NR4A3 as tumor suppressors in hematologic neoplasms. *Curr. Drug Targets* 16 38–46. 10.2174/1389450115666141120112818 25410408

[B41] Woolf A. D. Pfleger B. (2003). Burden of major musculoskeletal conditions. *Bull. World Health Organ.* 81 646–656. 14710506 PMC2572542

[B42] Yin Z. Chen X. Chen J. L. Shen W. L. Hieu Nguyen T. M. Gao L. (2010). The regulation of tendon stem cell differentiation by the alignment of nanofibers. *Biomaterials* 31 2163–2175. 10.1016/j.biomaterials.2009.11.083 19995669

[B43] You B. Jiang Y. Y. Chen S. Yan G. Sun J. (2009). The orphan nuclear receptor Nur77 suppresses endothelial cell activation through induction of IkappaBalpha expression. *Circ. Res.* 104 742–749. 10.1161/CIRCRESAHA.108.192286 19213954

[B44] Zhan Y. Du X. Chen H. Liu J. Zhao B. Huang D. (2008). Cytosporone B is an agonist for nuclear orphan receptor Nur77. *Nat. Chem. Biol.* 4 548–556. 10.1038/nchembio.106 18690216

[B45] Zhan Y. Y. Chen Y. Zhang Q. Zhuang J. J. Tian M. Chen H. Z. (2012). The orphan nuclear receptor Nur77 regulates LKB1 localization and activates AMPK. *Nat. Chem. Biol.* 8 897–904. 10.1038/nchembio.1069 22983157

[B46] Zhang Q. Lenardo M. J. Baltimore D. (2017). 30 Years of NF-kappaB: A blossoming of relevance to human pathobiology. *Cell* 168 37–57. 10.1016/j.cell.2016.12.012 28086098 PMC5268070

[B47] Zheng Z. Xiang S. Wang Y. Dong Y. Li Z. Xiang Y. (2020). NR4A1 promotes TNFalphainduced chondrocyte death and migration injury via activating the AMPK/Drp1/mitochondrial fission pathway. *Int. J. Mol. Med.* 45 151–161. 10.3892/ijmm.2019.4398 31746366 PMC6889925

[B48] Zhou L. Ruvolo V. R. McQueen T. Chen W. Samudio I. J. Conneely O. (2013). HDAC inhibition by SNDX-275 (Entinostat) restores expression of silenced leukemia-associated transcription factors Nur77 and Nor1 and of key pro-apoptotic proteins in AML. *Leukemia* 27 1358–1368. 10.1038/leu.2012.366 23247046 PMC3892989

