# Supplementary figures and images for: Reactivation of NR4A1 Restrains Chondrocyte Inflammation and Ameliorates Osteoarthritis in Rats
Source: Front Cell Dev Biol. 2020 Mar 17;8:158. doi: 10.3389/fcell.2020.00158 (PMC7090231; doi:10.3389/fcell.2020.00158)

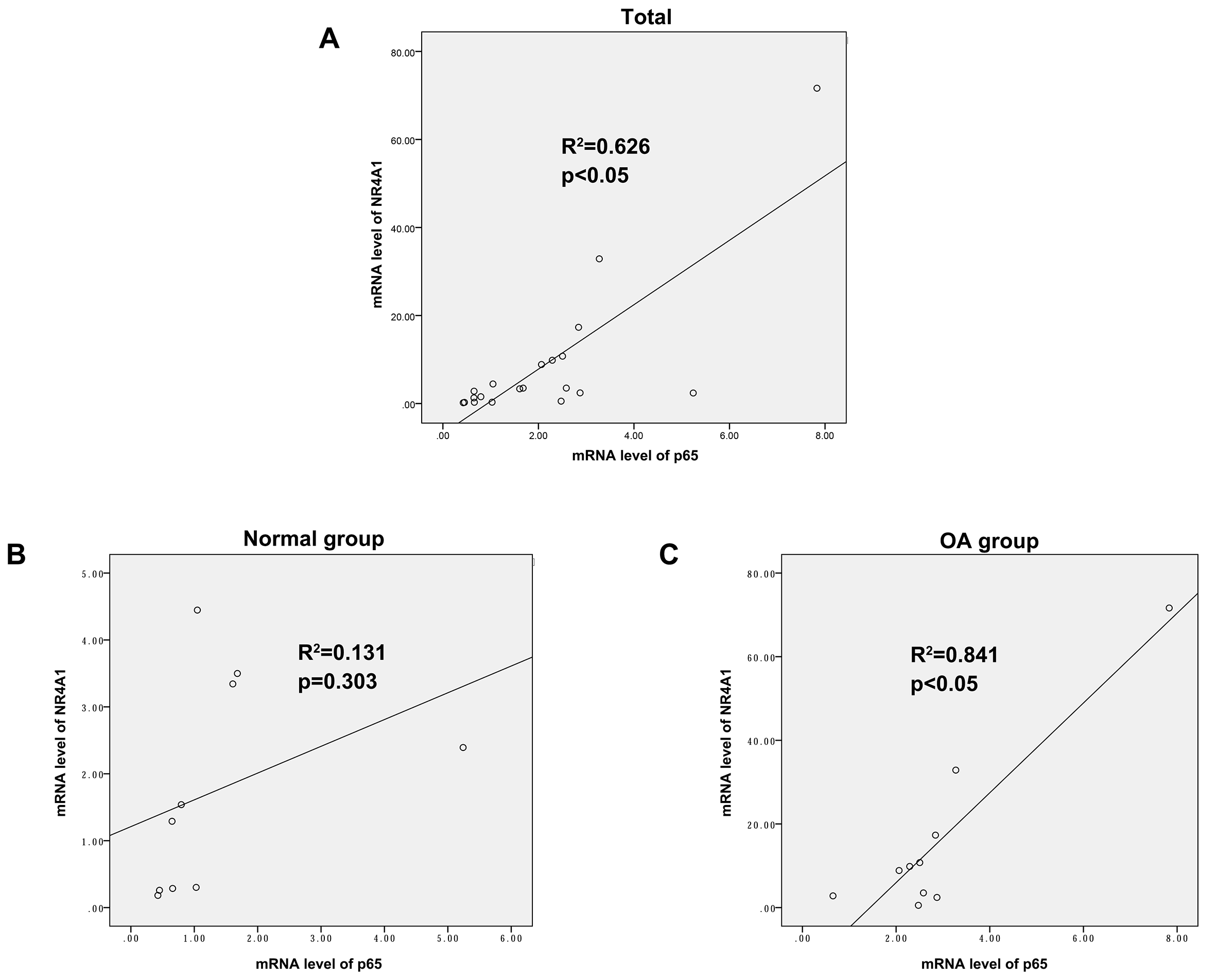

Supplement: FIGURE S1 — The correlation analysis of p65 expression and NR4A1 expression in human samples. (A) Total patients. (B) Normal group. (C) OA group. [file Image_1.TIF]

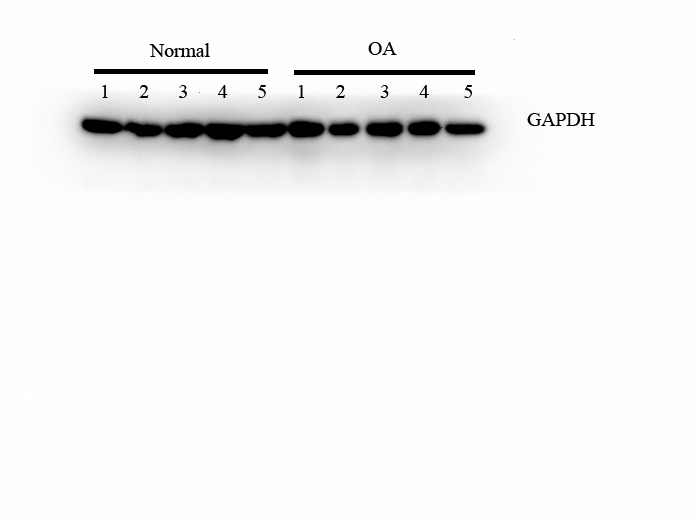

Supplement: Supplementary file 3 [file Data_Sheet_1.ZIP › original western-blot images/Fig.1 A GAPDH-1.jpg]

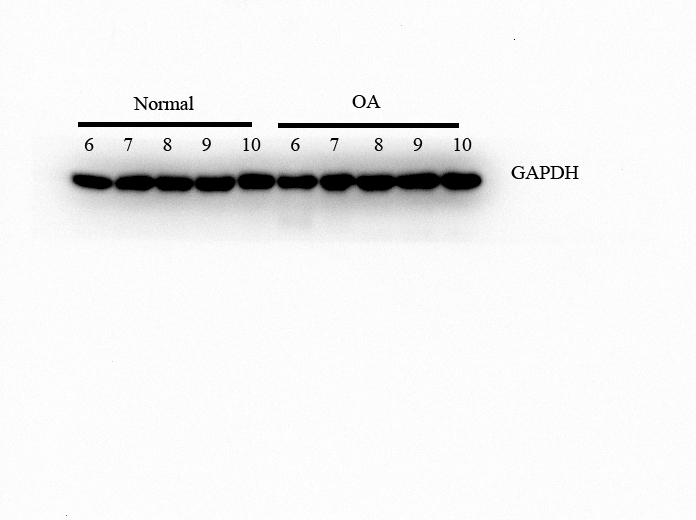

Supplement: Supplementary file 3 [file Data_Sheet_1.ZIP › original western-blot images/Fig.1 A GAPDH-2.jpg]

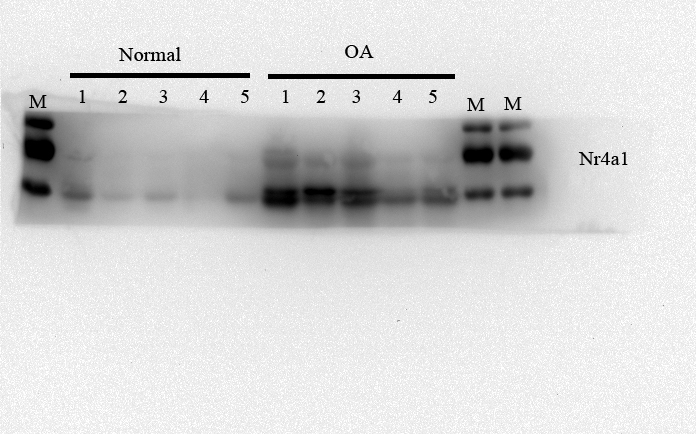

Supplement: Supplementary file 3 [file Data_Sheet_1.ZIP › original western-blot images/Fig.1 A Nr4a1-1.jpg]

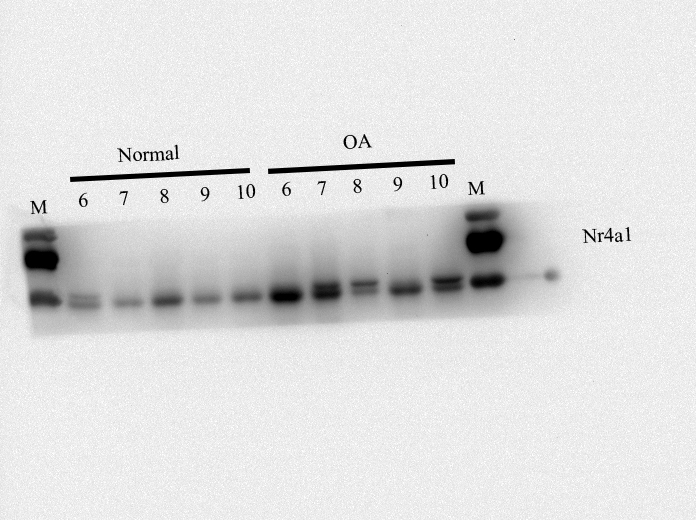

Supplement: Supplementary file 3 [file Data_Sheet_1.ZIP › original western-blot images/Fig.1 A Nr4a1-2.jpg]

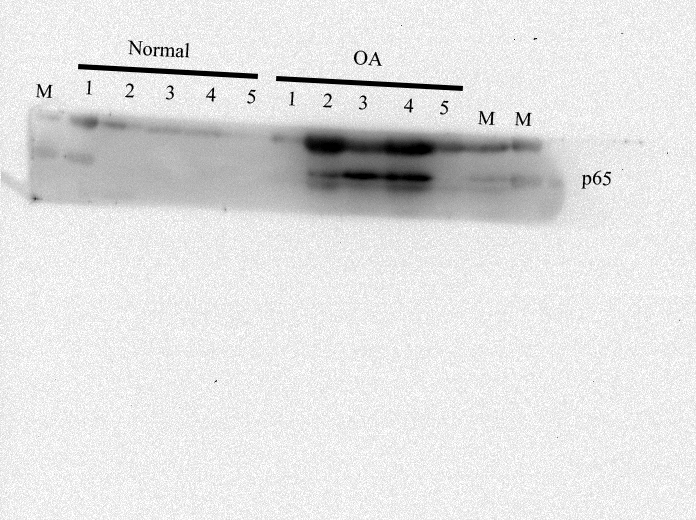

Supplement: Supplementary file 3 [file Data_Sheet_1.ZIP › original western-blot images/Fig.1 A p65-1.jpg]

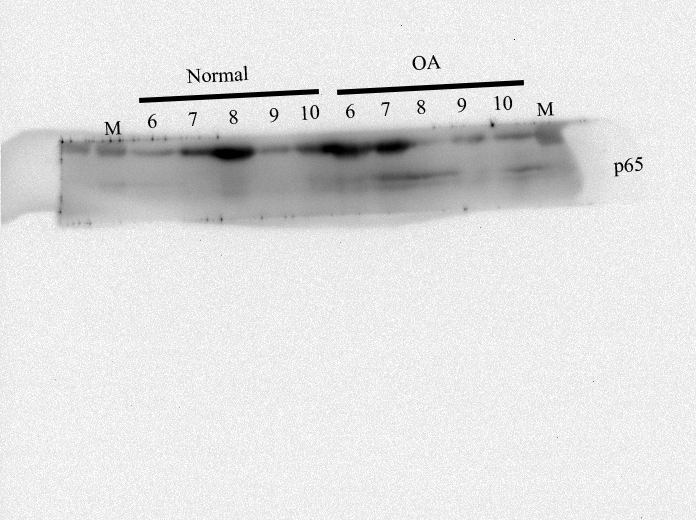

Supplement: Supplementary file 3 [file Data_Sheet_1.ZIP › original western-blot images/Fig.1 A p65-2.jpg]

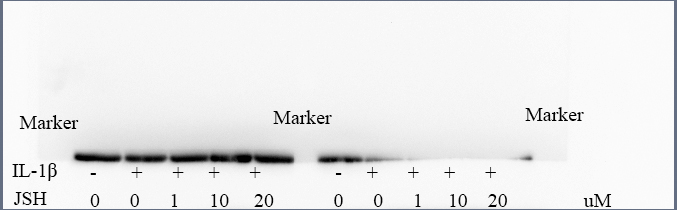

Supplement: Supplementary file 3 [file Data_Sheet_1.ZIP › original western-blot images/Fig.1 D actin.jpg]

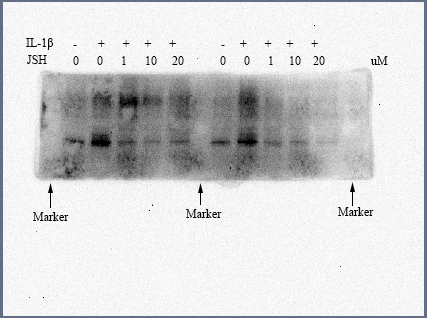

Supplement: Supplementary file 3 [file Data_Sheet_1.ZIP › original western-blot images/Fig.1 D Nr4a1.jpg]

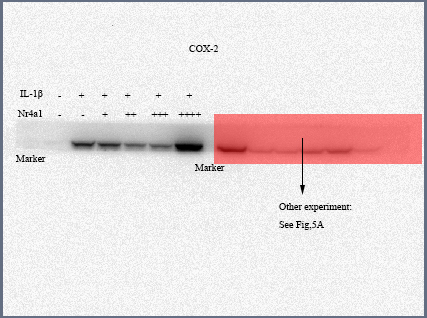

Supplement: Supplementary file 3 [file Data_Sheet_1.ZIP › original western-blot images/Fig.2 B cox-2.jpg]

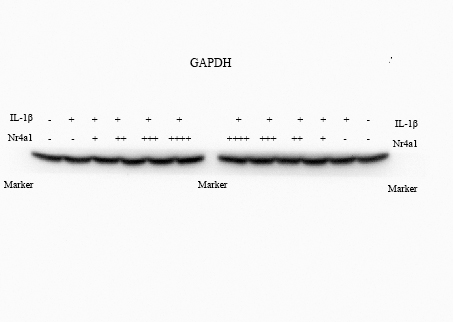

Supplement: Supplementary file 3 [file Data_Sheet_1.ZIP › original western-blot images/Fig.2 B GAPDH.jpg]

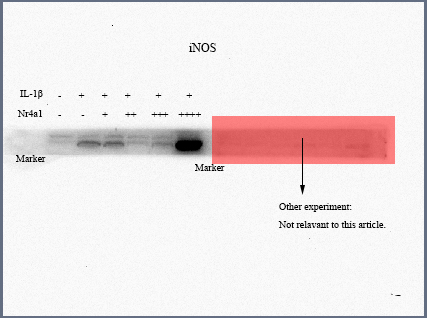

Supplement: Supplementary file 3 [file Data_Sheet_1.ZIP › original western-blot images/Fig.2 B iNOS.jpg]

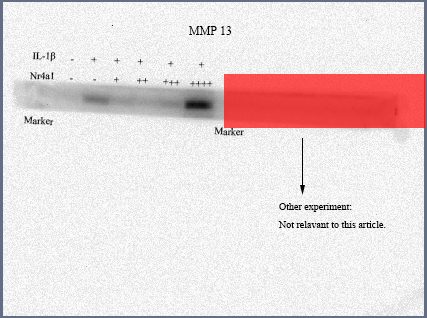

Supplement: Supplementary file 3 [file Data_Sheet_1.ZIP › original western-blot images/Fig.2 B MMP13.jpg]

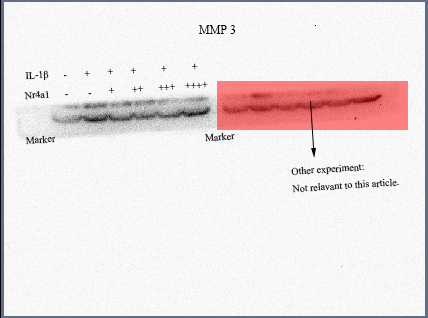

Supplement: Supplementary file 3 [file Data_Sheet_1.ZIP › original western-blot images/Fig.2 B MMP3.jpg]

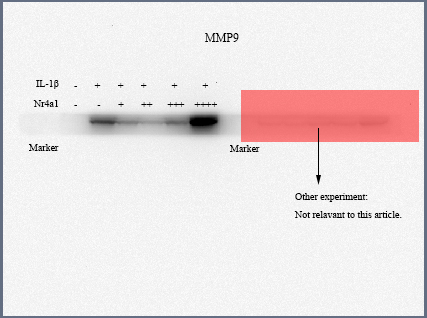

Supplement: Supplementary file 3 [file Data_Sheet_1.ZIP › original western-blot images/Fig.2 B MMP9.jpg]

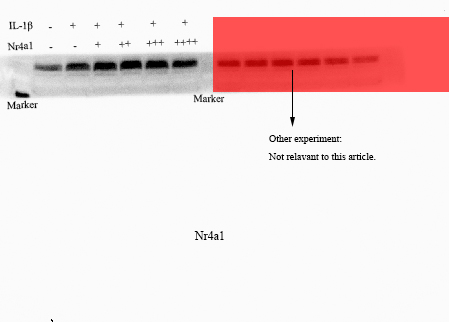

Supplement: Supplementary file 3 [file Data_Sheet_1.ZIP › original western-blot images/Fig.2 B Nr4a1.jpg]

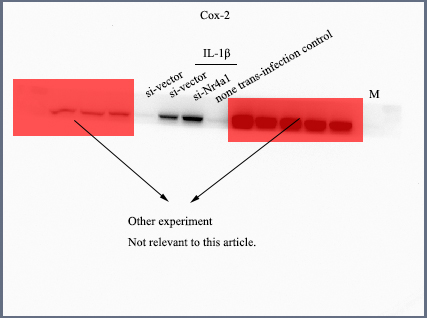

Supplement: Supplementary file 3 [file Data_Sheet_1.ZIP › original western-blot images/Fig.2 E cox2.jpg]

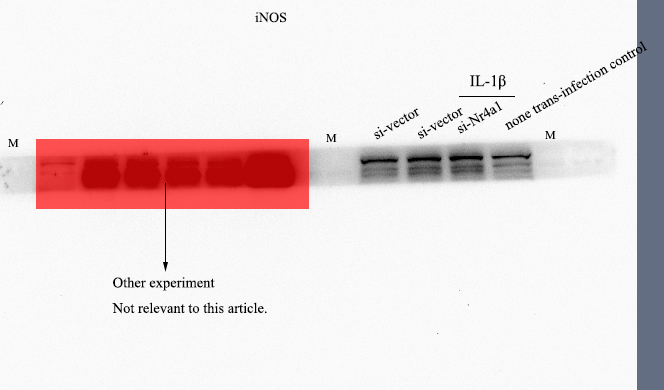

Supplement: Supplementary file 3 [file Data_Sheet_1.ZIP › original western-blot images/Fig.2 E iNOS.jpg]

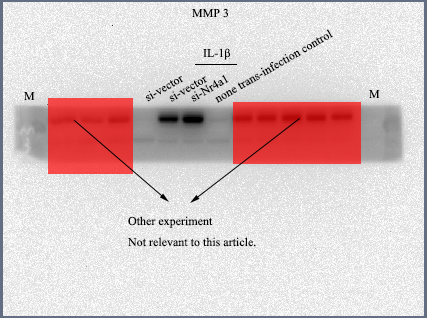

Supplement: Supplementary file 3 [file Data_Sheet_1.ZIP › original western-blot images/Fig.2 E MMP 3.jpg]

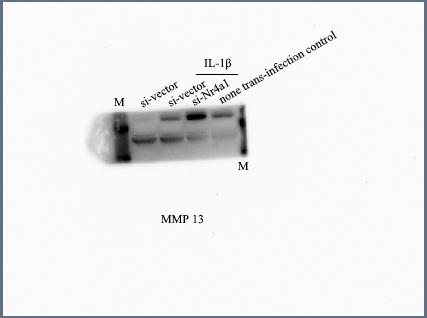

Supplement: Supplementary file 3 [file Data_Sheet_1.ZIP › original western-blot images/Fig.2 E MMP13.jpg]

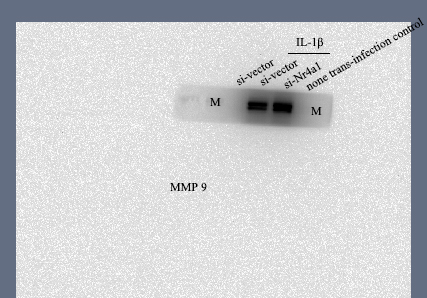

Supplement: Supplementary file 3 [file Data_Sheet_1.ZIP › original western-blot images/Fig.2 E MMP9.jpg]

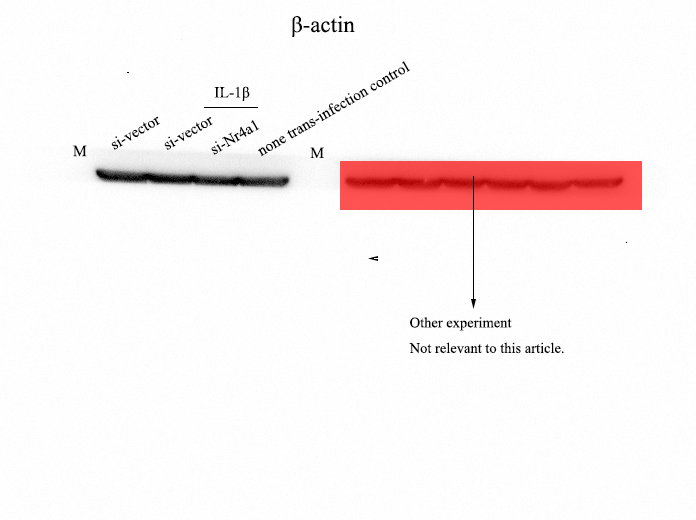

Supplement: Supplementary file 3 [file Data_Sheet_1.ZIP › original western-blot images/Fig.2 E Nr4a1 actin.jpg]

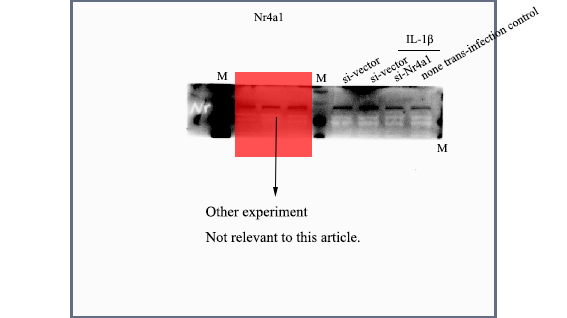

Supplement: Supplementary file 3 [file Data_Sheet_1.ZIP › original western-blot images/Fig.2 E Nr4a1.jpg]

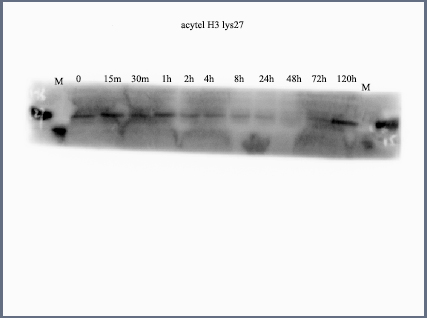

Supplement: Supplementary file 3 [file Data_Sheet_1.ZIP › original western-blot images/Fig.4 B acytel H3 lys27.jpg]

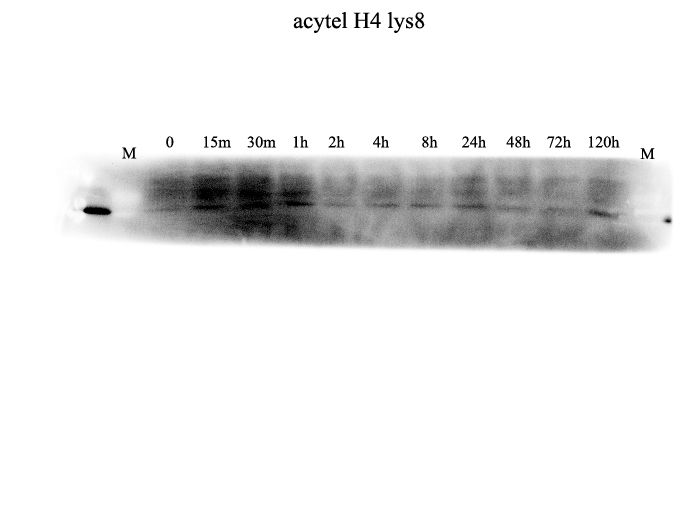

Supplement: Supplementary file 3 [file Data_Sheet_1.ZIP › original western-blot images/Fig.4 B acytel H4 lys8.jpg]

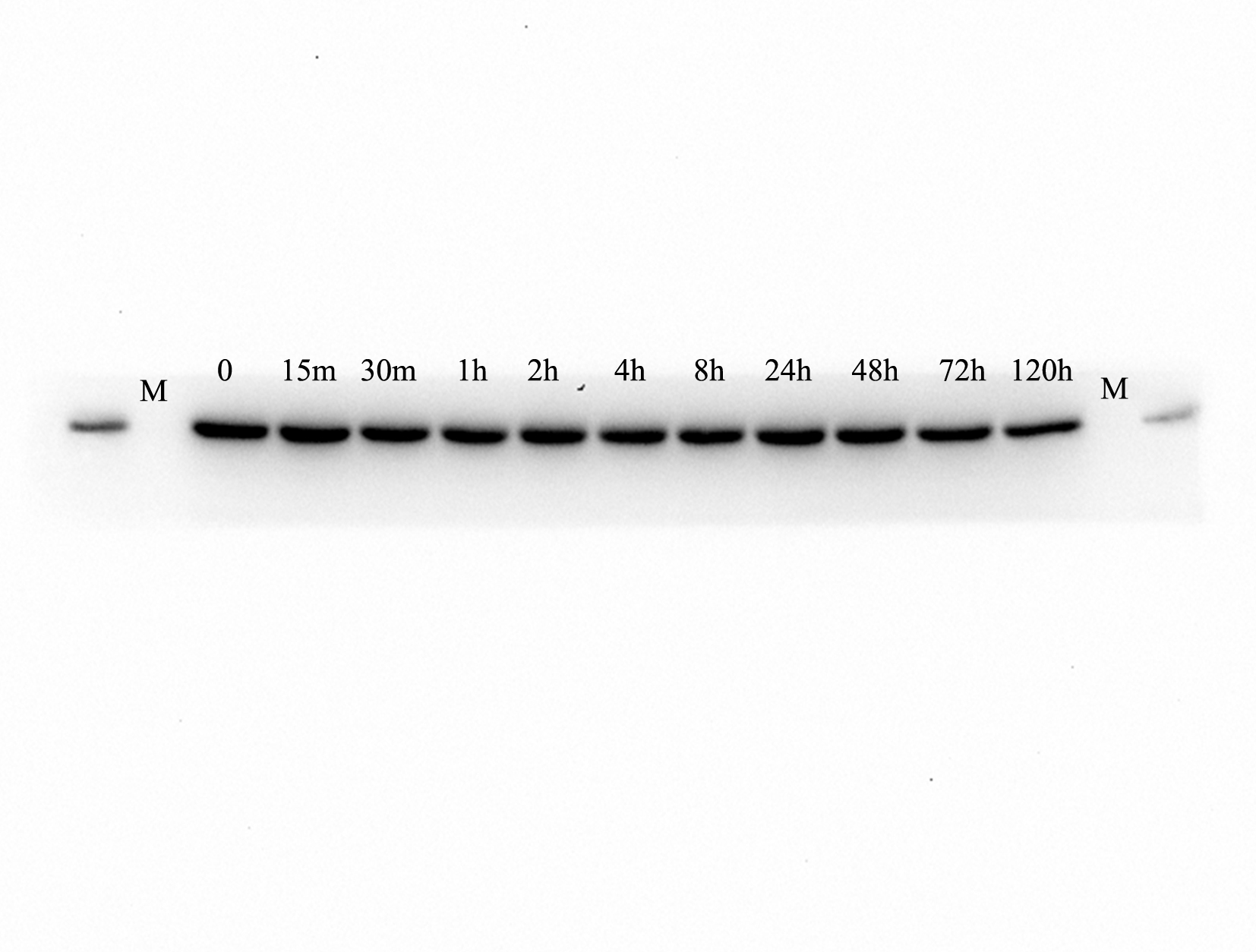

Supplement: Supplementary file 3 [file Data_Sheet_1.ZIP › original western-blot images/Fig.4 B GAPDH.jpg]

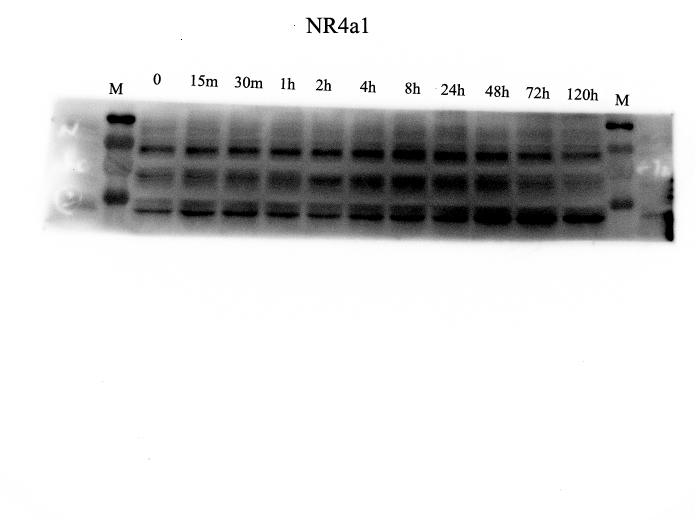

Supplement: Supplementary file 3 [file Data_Sheet_1.ZIP › original western-blot images/Fig.4 B Nr4a1.jpg]

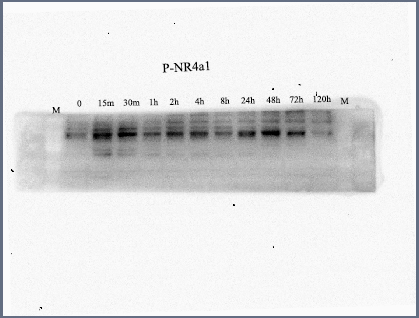

Supplement: Supplementary file 3 [file Data_Sheet_1.ZIP › original western-blot images/Fig.4 B p-Nr4a1.jpg]

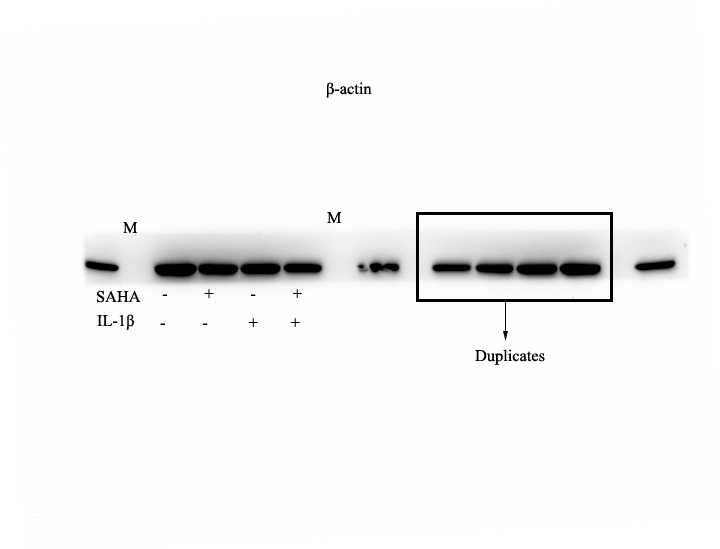

Supplement: Supplementary file 3 [file Data_Sheet_1.ZIP › original western-blot images/Fig.4 D actin.jpg]

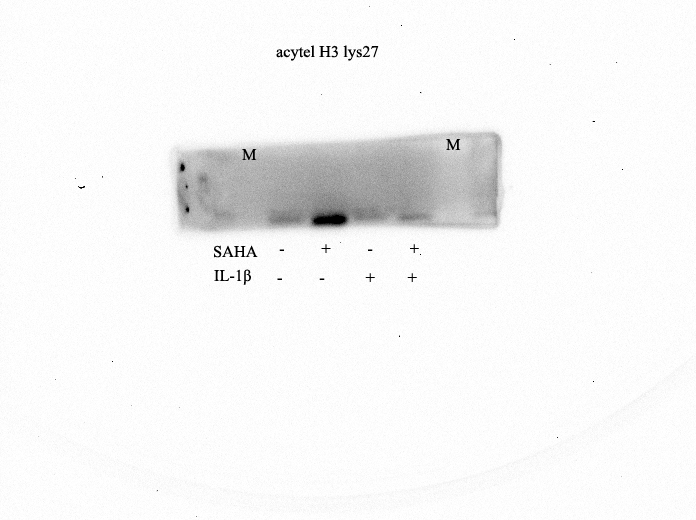

Supplement: Supplementary file 3 [file Data_Sheet_1.ZIP › original western-blot images/Fig.4 D acytel H3 lys27.jpg]

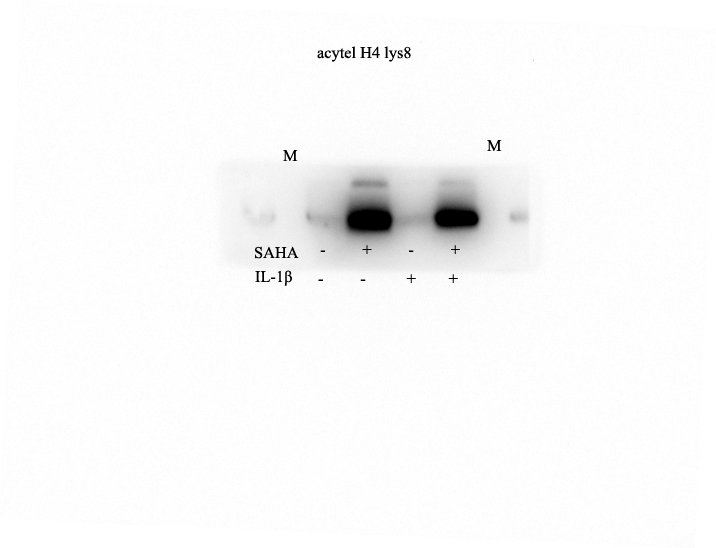

Supplement: Supplementary file 3 [file Data_Sheet_1.ZIP › original western-blot images/Fig.4 D acytel H4 lys8.jpg]

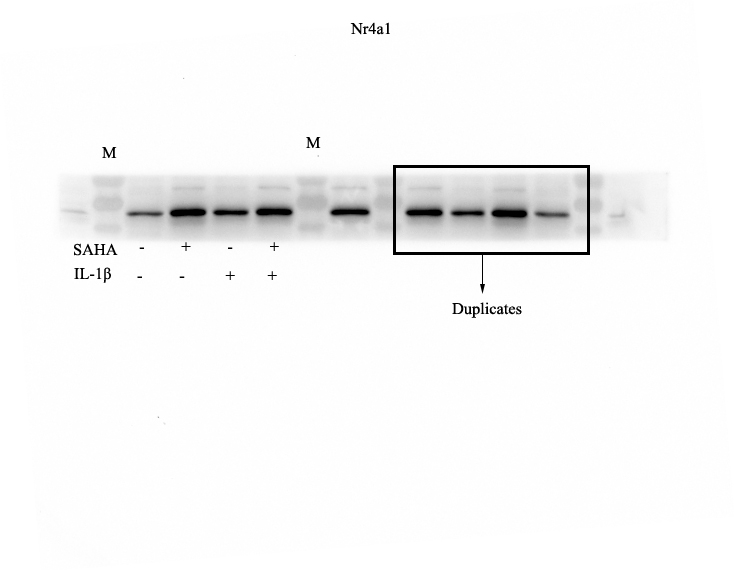

Supplement: Supplementary file 3 [file Data_Sheet_1.ZIP › original western-blot images/Fig.4 D Nr4a1.jpg]

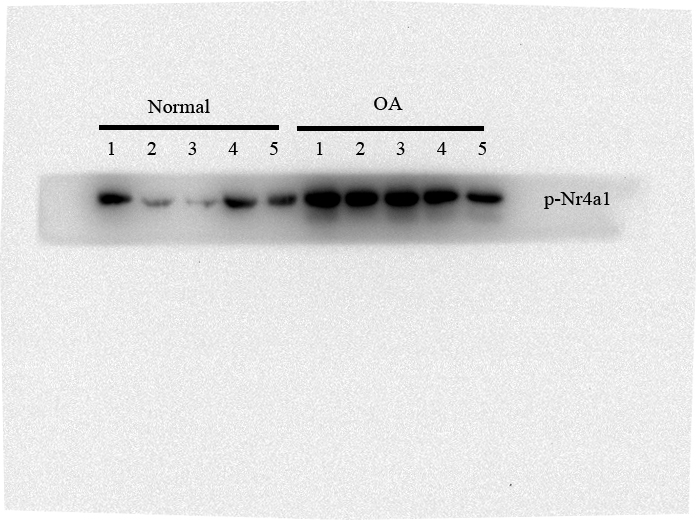

Supplement: Supplementary file 3 [file Data_Sheet_1.ZIP › original western-blot images/Fig.4 E p-Nr4a1-1.jpg]

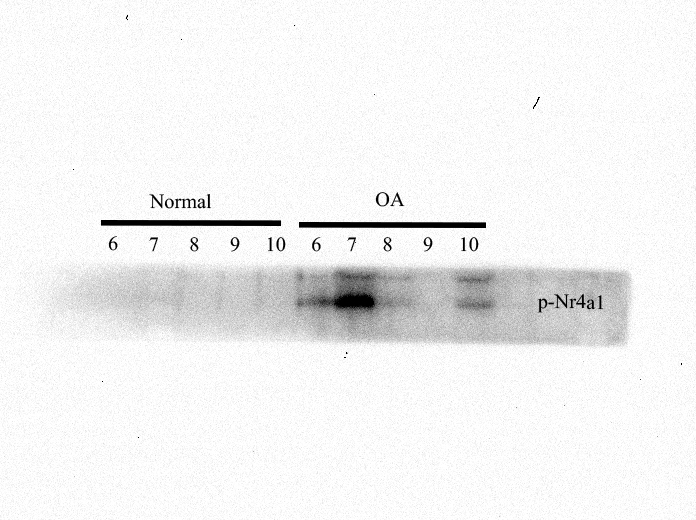

Supplement: Supplementary file 3 [file Data_Sheet_1.ZIP › original western-blot images/Fig.4 E p-Nr4a1-2.jpg]

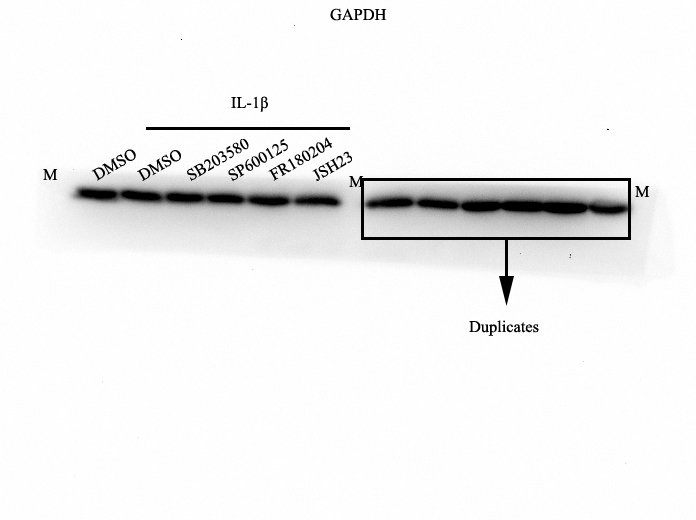

Supplement: Supplementary file 3 [file Data_Sheet_1.ZIP › original western-blot images/Fig.4 F GAPDH.jpg]

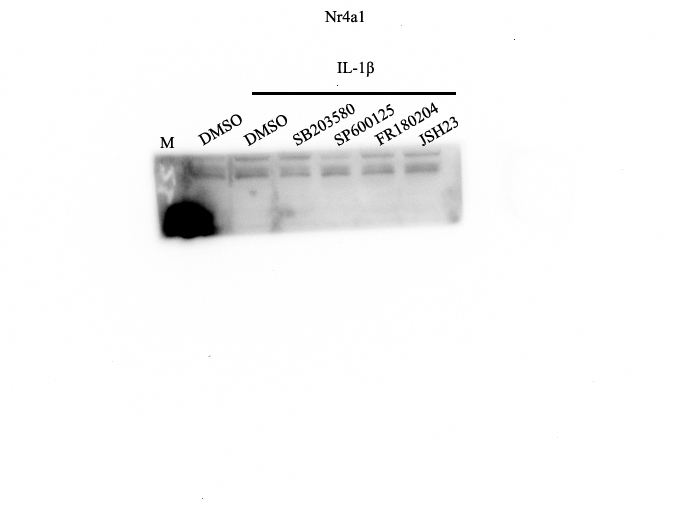

Supplement: Supplementary file 3 [file Data_Sheet_1.ZIP › original western-blot images/Fig.4 F Nr4a1.jpg]

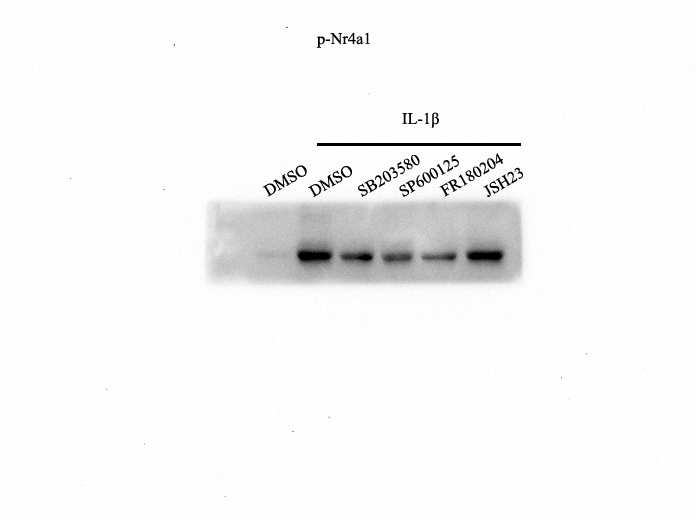

Supplement: Supplementary file 3 [file Data_Sheet_1.ZIP › original western-blot images/Fig.4 F p-Nr4a1.jpg]

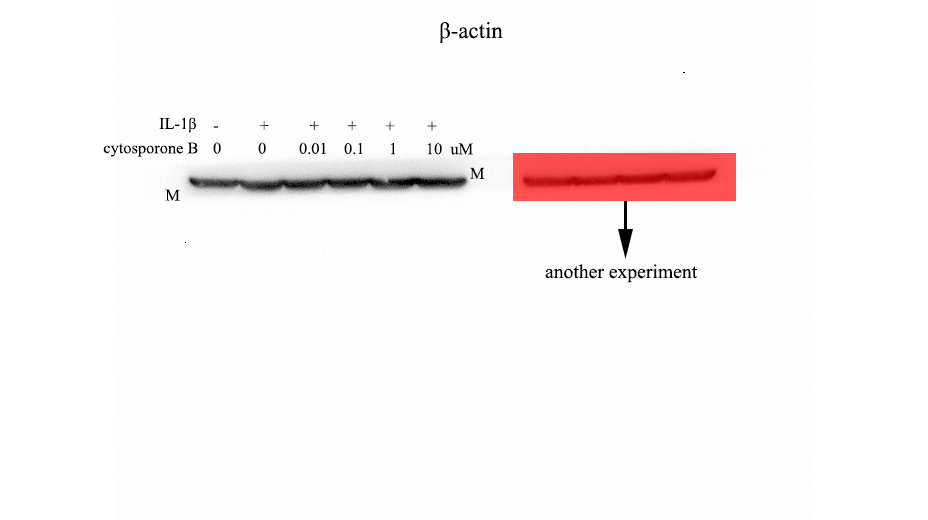

Supplement: Supplementary file 3 [file Data_Sheet_1.ZIP › original western-blot images/Fig.5 A actin.jpg]

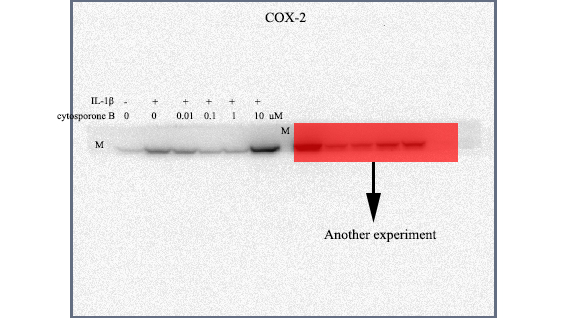

Supplement: Supplementary file 3 [file Data_Sheet_1.ZIP › original western-blot images/Fig.5 A cox2.jpg]

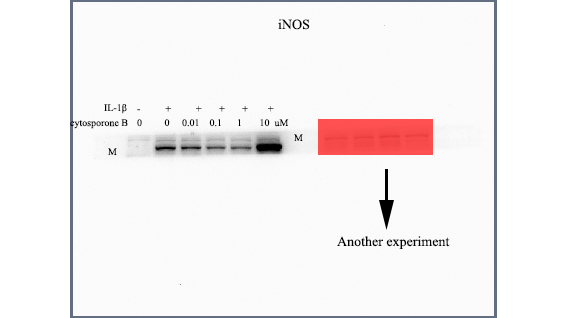

Supplement: Supplementary file 3 [file Data_Sheet_1.ZIP › original western-blot images/Fig.5 A iNOS.jpg]

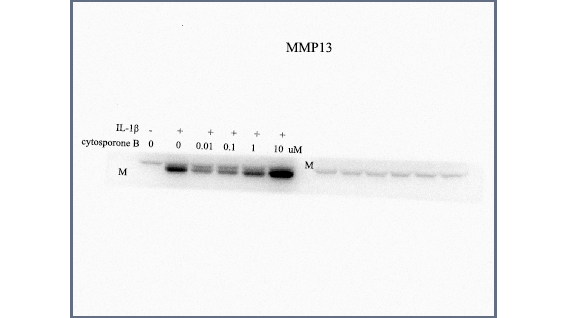

Supplement: Supplementary file 3 [file Data_Sheet_1.ZIP › original western-blot images/Fig.5 A MMP13.jpg]

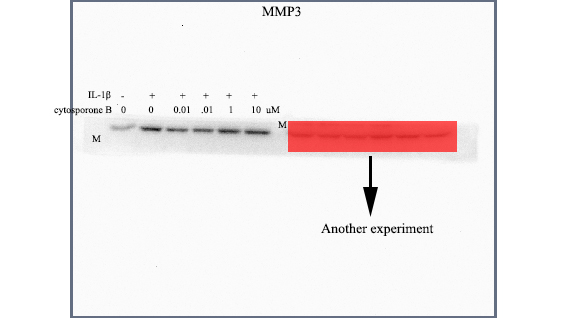

Supplement: Supplementary file 3 [file Data_Sheet_1.ZIP › original western-blot images/Fig.5 A MMP3.jpg]

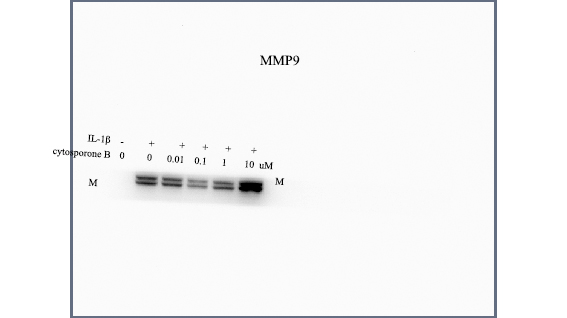

Supplement: Supplementary file 3 [file Data_Sheet_1.ZIP › original western-blot images/Fig.5 A MMP9.jpg]

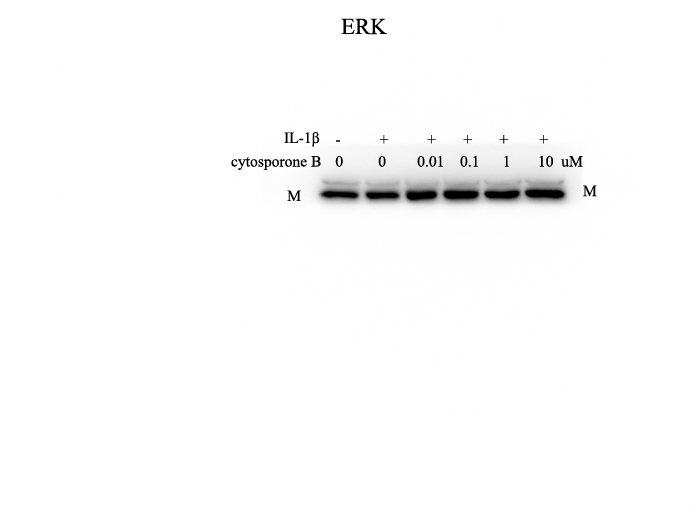

Supplement: Supplementary file 3 [file Data_Sheet_1.ZIP › original western-blot images/Fig.5 G ERK.jpg]

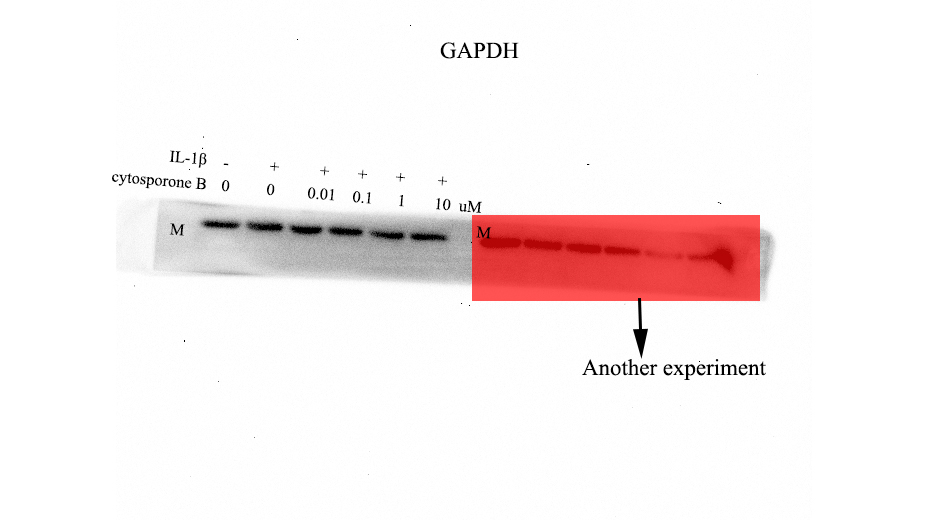

Supplement: Supplementary file 3 [file Data_Sheet_1.ZIP › original western-blot images/Fig.5 G GAPDH.jpg]

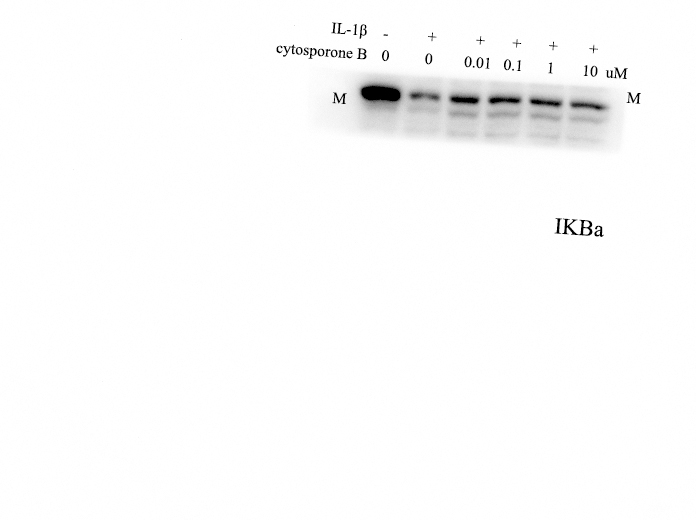

Supplement: Supplementary file 3 [file Data_Sheet_1.ZIP › original western-blot images/Fig.5 G IKba.jpg]

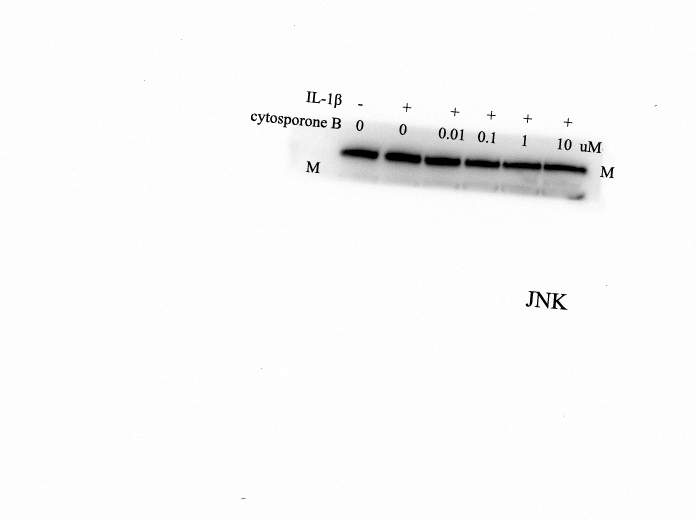

Supplement: Supplementary file 3 [file Data_Sheet_1.ZIP › original western-blot images/Fig.5 G JNK.jpg]

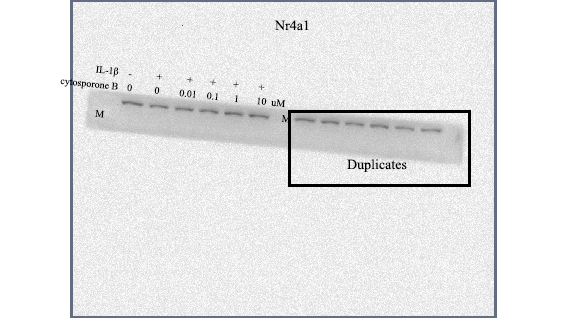

Supplement: Supplementary file 3 [file Data_Sheet_1.ZIP › original western-blot images/Fig.5 G Nr4a1.jpg]

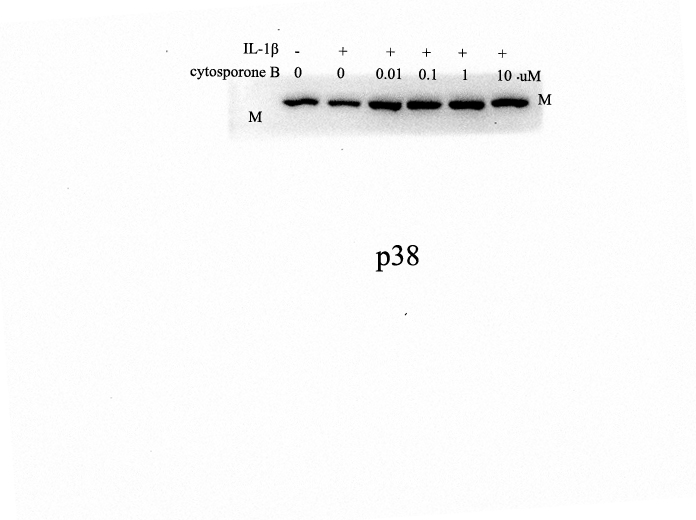

Supplement: Supplementary file 3 [file Data_Sheet_1.ZIP › original western-blot images/Fig.5 G p38.jpg]

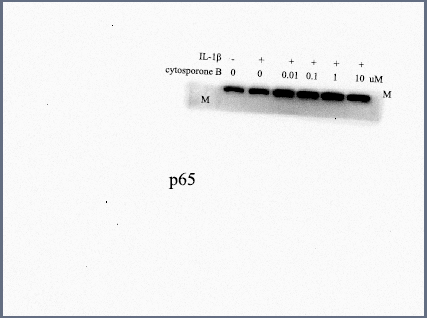

Supplement: Supplementary file 3 [file Data_Sheet_1.ZIP › original western-blot images/Fig.5 G p65.jpg]

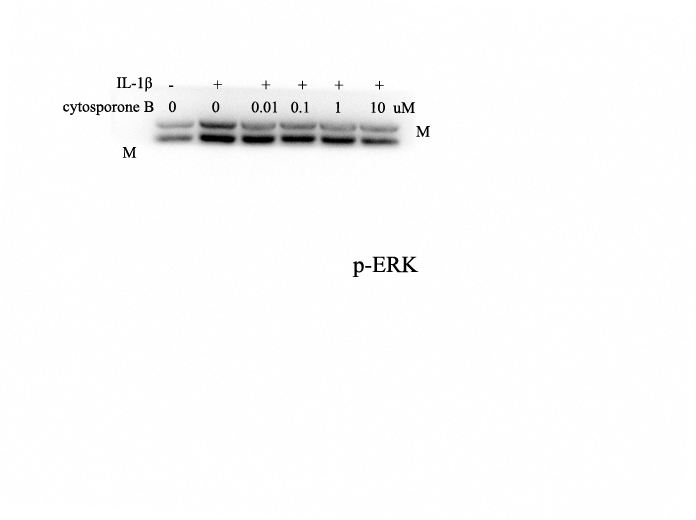

Supplement: Supplementary file 3 [file Data_Sheet_1.ZIP › original western-blot images/Fig.5 G p-ERK.jpg]

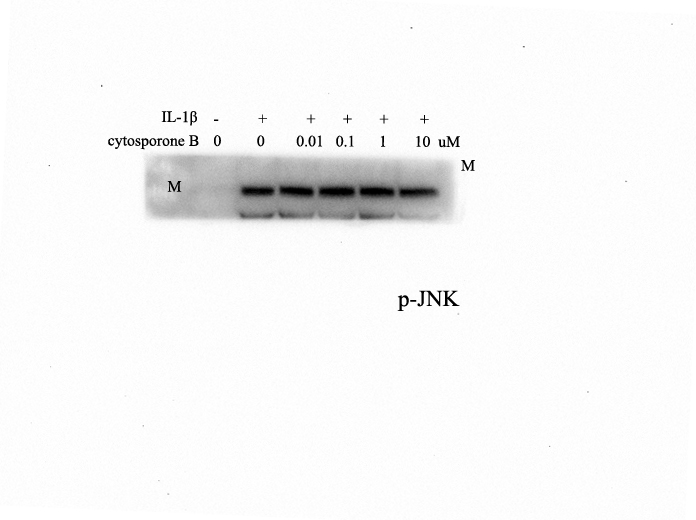

Supplement: Supplementary file 3 [file Data_Sheet_1.ZIP › original western-blot images/Fig.5 G p-JNK.jpg]

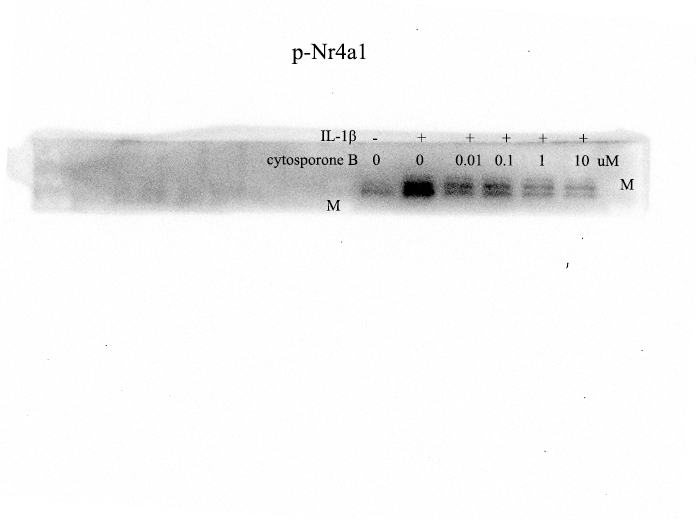

Supplement: Supplementary file 3 [file Data_Sheet_1.ZIP › original western-blot images/Fig.5 G p-Nr4a1.jpg]

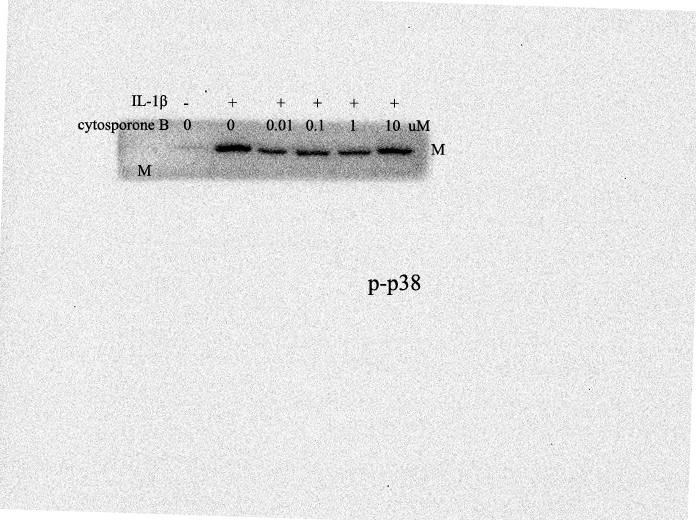

Supplement: Supplementary file 3 [file Data_Sheet_1.ZIP › original western-blot images/Fig.5 G p-p38.jpg]

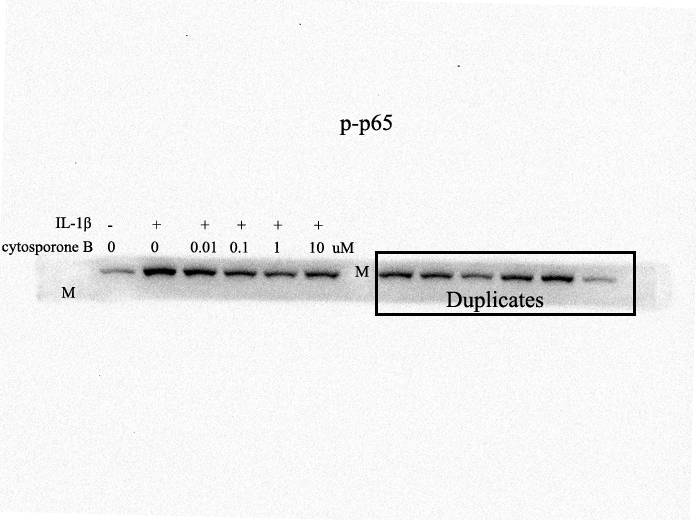

Supplement: Supplementary file 3 [file Data_Sheet_1.ZIP › original western-blot images/Fig.5 G p-p65.jpg]
