# Supplementary material for: Reactivation of NR4A1 Restrains Chondrocyte Inflammation and Ameliorates Osteoarthritis in Rats
Source: Front Cell Dev Biol. 2020 Mar 17;8:158. doi: 10.3389/fcell.2020.00158 (PMC7090231; doi:10.3389/fcell.2020.00158)
Supplement: TABLE S1 — Primer sequences used in this study. [file Table_1.docx]

Supplementary Table 1. Primer sequences used in this study.

| Gene | Forward | Reverse |
| --- | --- | --- |
| Human NR4A1 | GCTACCTTCAAAACCCAAGCAG | AACCGGAGAGCAGGTCGTAG |
| Human p65 | AGGCTATCAGTCAGCGCATC | CATTCAGGTCGTAGTCCCCAC |
| Human 18S | CCTGAGAAACGGCTACCACA | ACCAGACTTGCCCTCCAATG |
| Rat NR4A1 | GGCTACCTTCAAAACCCAAGC | GGAGCCAGAGAGCAAGTCAT |
| Rat MMP3 | CAGGCATTGGCACAAAGGTG | GTGGGTCACTTTCCCTGCAT |
| Rat MMP9 | GGATAACGAGTTCTCTGGCGT | CTGCAGGAGGTCATAGGTCAC |
| Rat MMP13 | GCAAACCCTGCGTATTTCCAT | GATAACCATCCGAGCGACCTTT |
| Rat COX2 | GATGTTCGCATTCTTTGCCCA | TACACCTCTCCACCGATGAC |
| Rat iNOS | CCTTACGAGGCGAAGAAGGACAG | CAGTTTGAGAGAGGAGGCTCCG |
| Rat HDAC1 | ACATGCCAAGTGTGTGGAGT | TGTGAAGCTTGAAATCTGGTCC |
| Rat HDAC2 | GCTGGGCTGCTTCAACCTAA | ACGTCCAACATCGAGCAACA |
| Rat HDAC3 | AATGTGCCCTTACGGGATGG | TTCCCCATGTCCTCGAATGC |
| Rat HDAC4 | CTCCAGCAGAGGTTGAATGTG | TTGACATTGAAACCCACGCCT |
| Rat HDAC5 | GGTCGTAAAGCCACACTGGA | TCCAGCTTCTGCCGGTTAAG |
| Rat HDAC6 | ATGCCCAAGTCATTGCTGGA | CTCGCCCTACTTGGCTACTG |
| Rat HDAC7 | GGGTGCTCTACATATCCCTTCA | GCAATGGGCATCACCACTATC |
| Rat HDAC9 | ACGGCCAAATACTTGAGAGACC | CCTTCTCCCAGACCTACTCCAA |
| Rat HDAC10 | TATAGCAGCCAGACATGCCA | GTTTCCATGCTCATAGCGGTG |
| Rat 18S | CCTGAGAAACGGCTACCACA | ACCAGACTTGCCCTCCAATG |
